# Unpacking Neighborhood Socioeconomic Status in Children’s Health Research from an Environmental Justice Perspective: A Scoping Review

**DOI:** 10.1007/s40572-024-00445-8

**Published:** 2024-04-10

**Authors:** Ananya Bhaktaram, Amii M. Kress, Zone Li, Emily A. Knapp

**Affiliations:** 1 Department of Health, Behavior and Society, Johns Hopkins University, 615 N. Wolfe St, Baltimore, MD 21205 USA; 2 Department of Epidemiology, Johns Hopkins University, Baltimore, MD USA

**Keywords:** Social epidemiology, Neighborhoods, Socioeconomic status, Environmental justice, Health disparity, Child health and development

## Abstract

**Purpose of Review:**

Clearly defining and measuring neighborhood socioeconomic status (nSES) is a key first step in achieving environmental justice, as the disproportionate distribution of environmental hazards and access to resources is heavily influenced by socioeconomic factors. This scoping review explores the definition of neighborhoods, measurement of neighborhood socioeconomic status (nSES), and studies that evaluated the association between nSES and child health in accordance with PRISMA guidelines.

**Recent Findings:**

We identified 4112 articles published on US pediatric populations between 2013 and 2022. We identified 170 distinct indicators across seven broad domains of nSES used to create 121 different measures of nSES across the 206 publications included in this review. While there is considerable interest in nSES and children’s health, there is also substantial variation in the measurement of neighborhood as a geographic unit and nSES as a construct.

**Summary:**

We observed methodological challenges related to the identification of neighborhood boundaries, indicator selection, and nSES measure definition(s). We discuss common pitfalls in neighborhood research that can complicate identifying, targeting, and resolving environmental injustices. Lastly, we put forward a series of recommendations to reduce measurement error and improve inference, in addition to reporting recommendations for neighborhoods and health research that can aid in improving our understanding of pathways between neighborhood context and child health, inform policy development, and allocate resources to achieve environmental justice.

**Supplementary Information:**

The online version contains supplementary material available at 10.1007/s40572-024-00445-8.

## Introduction

Socioeconomic status (SES) during childhood is a powerful predictor of health, health-related behaviors, and well-being across the life course [1, 2]. SES is a complex social phenomenon typically characterized along three domains: education, employment, and income [3]. Neighborhood SES (nSES) refers to the physical, social, and economic positioning of the environment in which people live [4, 5]. Neighborhood SES is a key construct for understanding environmental health and promoting environmental justice. Low SES neighborhoods are disproportionately affected by air pollution and environmental toxins, and low SES persons and residents of low SES neighborhoods are especially vulnerable to the effects of these environmental stressors [6, 7]. Neighborhoods define the socioeconomic, physical, and political contexts that mediate environmental exposures and children’s health-related behaviors and outcomes [8, 9]. Recent studies show a dose-response relationship to nSES independent of individual SES: the longer a child resides in a higher SES neighborhood, regardless of their household SES, the more significant the impact on their health outcomes [10, 11]. Thus, neighborhoods represent a unique geographic unit that captures physical and built exposures, socioeconomic positioning, and social interactions between residents.

Disentangling the effects of nSES and environmental exposures requires careful design and measurement of both constructs [12•, 13]. Additionally, it is important to understand the effects of nSES on children and adolescents as children and adolescents are influenced by the neighborhoods in which they live, play, and learn [5, 14, 15]. 

Early life nSES is biologically embedded, as childhood is a sensitive period when residential environmental exposure has stronger effects on development that can be amplified over time [16, 17]. NSES is a key determinant of child health and well-being [18]. However, if we do not have a clear understanding of how nSES is conceptualized and operationalized, we cannot provide clear guidance for developing policies to modify this crucial aspect of the environment [19, 20]. For example, the strength of associations between different nSES components and health may lead to the prioritization of different types of interventions, or the size of the geospatial area used in assessing health outcomes may impact policy recommendations for improving childhood health [21••].

A recent review of the environmental justice literature found that 60% of environmental justice questions related to SES [12•]. However, SES and nSES specifically remain ambiguously defined across much of the public health literature. Nearly a decade after van Vuuren et al. reviewed neighborhood socioeconomic deprivation in child health studies, there is still no established set of recommendations for defining and operationalizing nSES [22]. A handful of smaller reviews have explored nSES effects on child health but did not evaluate nSES measures [23–27]. Van Vuren reported that most studies do not give a theoretical justification for which domains or indicators are appropriate for nSES measures but did not provide focused recommendations and guidelines [22]. Another review from Arcaya et al. focused more broadly on neighborhood effects, of which nSES was the most studied, and focused on the definition of neighborhoods and analytical methods but gave no special consideration to child health [28].

The goal of this scoping review is to update and build upon the van Vuuren and Arcaya reviews, and to describe the current state of nSES literature for environmental health researchers [22, 28]. We expand upon these reviews by including studies published after February 1, 2013, and focusing on the measurement of nSES in children’s health research in the USA. We also use these findings to inform recommendations for measuring nSES and provide a comprehensive catalogue of established nSES measures.

## Methods

### Search Strategy

To identify relevant studies that explore the association between nSES, and child health published between February 1, 2013, and August 31, 2022, we performed a literature search in PubMed. The search was restricted to studies published after February 1, 2013, to include all studies not covered by previous reviews [22, 28]. We found 50 relevant articles via a manual Google Scholar search to develop the search strategy. We created a search strategy in PubMed using the title, abstract, keyword, and medical subject headings (MeSH) terms presented in Table 1.

**Table 1:** Search terms

Concept number	MeSh term and keywords	Results
#1	Area Deprivation Index[tw]	488
#2	**“Residence Characteristics” [Mesh]** OR residence socioeconomic characteristic*[tw] OR residential socioeconomic characteristic*[tw] OR neighborhood characteristic*[tw] OR neighborhood context*[tw] OR neighborhood condition*[tw] OR neighborhood level[tw] OR census block*[tw] OR zip code*[tw] OR “area deprivation index” [tw]	83,829
#3	**“Socioeconomic Factors” [Mesh]** OR “Socioeconomic Factor*” [tw] OR “Socioeconomic Status*” [tw] OR SES [tw] OR socioeconomic characteristic*[tw]	546,491
#4	**“Infant” [Mesh] OR “Adolescent” [Mesh] OR “Minors” [Mesh] OR “Young Adult” [Mesh] OR “Child” [Mesh]** OR adolescen*[tw] OR teen*[tw] OR youth*[tw] OR young adult*[tw] OR juvenile*[tw] OR child*[tw] OR infant*[tw] OR newborn*[tw] OR perinatal*[tw] OR “life course”[tw]	5,174,961
#5	**“Health” [Mesh]** OR health*[tw] OR disparity[tw] OR disparities[tw]	4,628,758
#6	index[tw] OR indices[tw] OR survey[tw] OR surveys[tw] OR data[tw]	7,550,394
#7	#1 OR (#2 AND #3 AND #4 AND #5 AND #6) Published between February 1, 2013, and August 31, 2022.	4,112

### Inclusion Criteria

The focus of this study was characterizing neighborhood-level, objective, multi-indicator measures of nSES in child health research in the USA. Inclusion criteria included published in English, US setting, child health outcome (inclusive of perinatal and birth outcomes), included an objective, multi-indicator neighborhood or area-level SES measure (excluding geographies of county and larger), and had a sample size of at least 100 participants.

Given the multi-dimensional nature of nSES, as SES is traditionally characterized along at least three domains: income, education, and employment, and the availability of area-level data, we believe that there is not a good reason to solely rely on single-indicator measures of nSES [3, 29••]. Thus, we excluded single-indicator measures of nSES like census tract poverty level. We limited our search to articles with US populations because data availability, administrative units, and theoretical considerations for the measurement of nSES vary by country. We excluded reviews, abstracts, meta-analyses, book chapters, dissertations, posters, and commentaries.

**Fig. 1:**
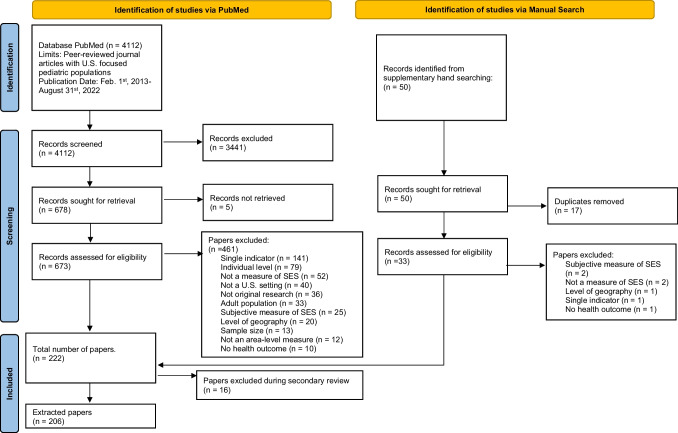
Data search and inclusion (flowchart). Adapted from Page MJ, McKenzie JE, Bossuyt PM, Boutron I, Hoffmann TC, Mulrow CD, et al. The PRISMA 2020 statement: an updated guideline for reporting systematic reviews. BMJ 2021;372: n71. doi: 10.1136/bmj. n71

Our study screening and data extraction process is presented in Fig. 1.

### Study Selection

Two reviewers (AB and ZL) conducted the literature search, study selection, and data extraction. Covidence was used to screen and extract articles based on the inclusion/exclusion criteria outlined [30]. Inter-reviewer reliability was established by beta-testing data with 15 articles to ensure consistency among the two extractors. A third reviewer adjudicated discrepancies (EK). All articles underwent dual review through the screening stage. A high level of consensus (90%) among our reviewers during our test phase informed us of our decision to move forward with a single extraction of the included articles, followed by a secondary review of all extracted materials by AB.

### Data Extraction

We extracted the following information: author(s), title, year, nSES domains (i.e., income, education, employment, marital status, housing, transportation, other), nSES indicators, data source(s), geographic unit, health outcome, age categories (e.g., perinatal/infancy, early childhood), and type of population (e.g., general population, clinical cohort). We extracted data on the selection of established nSES measures and/or methodological development of new study-specific measures. Free text notes about the measure were also included. We extracted information on the type of measure and data source(s) used in each measure. We grouped health outcomes into 10 categories. All studies that met the inclusion criteria were extracted using a data extraction tool built in Covidence and then exported into Excel for analysis [30]. Reporting of this review conforms with the guidelines set by the PRISMA extension for scoping reviews [31].

We grouped all indicators included in each nSES measure under the seven nSES domains for this review (i.e., income, education, employment, marital status, housing, transportation, other). We chose to include transportation as a nSES domain because transportation infrastructure and public transit access are unequally distributed environmental exposures as well as social and economic consequences [32]. Additionally, many indicators included in the “other” domain are not measures of nSES, such as air pollution, proximity to superfund sites, the number of summer days with maximum temperature above 90F but were extracted for this review because the studies incorporated these indicators into their nSES measures and were environmental justice indicators. Extracting these elements allows us to comment on measurement creation and variability more completely.

## Results

### Search Results

Our PubMed search found 4112 articles published between February 1, 2013, to August 31, 2022 (Fig. 1). All articles underwent title and abstract review, at which point 3441 articles that did not meet the inclusion criteria were excluded. Five articles were unavailable for full-text review due to broken links or inaccurate DOI numbers. Of the 673 articles that underwent full-text review, we excluded 461 articles, most commonly because 1) the study was outside of the USA (*n* = 40); 2) the level of geographic analysis was at the county, state, or regional level (*n* = 24); 3) the study used a single-indicator measure of nSES (*n* = 146); 4) the study used a subjective measure of nSES (*n* = 29); or 5) was not an original research paper (*n* = 37). We excluded 32 studies during the full-text review that solely used racial composition as a proxy for nSES. Our PubMed search was supplemented by hand searching, which identified 17 additional articles. In total, 222 articles were extracted. During the secondary review of the extracted data, we excluded 16 additional articles. Ultimately, there were 206 extracted articles in our final sample.

#### nSES Measures

##### Existing or New Measures of nSES

Of the 206 studies included, 109 (52.9 %) used established measures of nSES, most commonly the area deprivation index (ADI) (52 studies). There were 121 unique measures of nSES: 24 established and 97 study-specific measures (Table 2). Income was the most frequently represented domain included in 202 (98.1%) studies and 118 (97.5%) measures (Table 2). Education was the second most incorporated domain, with 174 (84.5%) studies and 95 (78.5%) measures. A total of 172 (83.5%) studies and 92 (76.0%) measures included an employment indicator. Transportation was the least represented domain, with 93 (45.1%) studies and 13 (10.7%) measures. Additionally, 128 (62.1%) studies and 61 (50.4%) measures incorporated indicators that did not fit into one of our six primary domains, such as the average number of superfund sites within a two-mile radius and the crime rate (Supplementary Table 1). Seventy-four articles incorporated a racial and/or ethnic composition indicator into their measure of nSES.

**Table 2:** Characteristics of neighborhood socioeconomic (nSES) measures

	Studies, *N* (%)	nSES measures, *N* (%)
Number^1^	**206**	**121**^**2**^
Measure type		
Existing	**109**	**24**
Study specific	**97**	**97**
Indicator domains		
Income	202 (98.1)	118 (97.5%)
Education	174 (84.5)	95 (78.5%)
Employment	172 (83.5)	92 (76.0%)
Marital status	131 (63.6%)	62 (51.2%)
Housing	141 (68.4%)	62 (51.2%)
Transportation	93 (45.1%)	13 (10.7%)
Other	128 (62.1%)	61 (50.4%)
Measure creation method		
Index^3^	158 (76.7%)	77 (63.6%)
Latent variable method^4^	19 (9.2%)	19 (15.7%)
Data-driven reduction^5^	37 (18.0%)	32 (26.4%)
Data source		
American Community Survey	122 (59.2%)	47 (38.8%)
Decennial Census	76 (36.9%)	61 (50.4%)
Other^6^	32 (15.5%)	29 (23.9%)
Geography^7^		
Census block groups	49 (23.8%)	
Census tracts	119 (57.8%)	
Euclidean distance around home	1 (0.5%)	
School census tracts^8^	3 (1.5%)	
Zip code	34 (16.5%)	
Other	10 (4.9%)	
Unspecified	3 (1.5%)	

^1^ Response categories will not sum up to 206 and 121 for type of measure, data source used, and level of geographic analysis, as they were extracted using a check all that apply response option

^2^ Twenty-four established measures and 97 study-specific measures

^3^ An index refers to a composite score created by a summed average

^4^ Latent variable methods include theory-driven measurement scales of latent constructs from observed indicators, such as confirmatory factor analysis and latent class analysis

^5^ Data-driven reduction methods refer to dimensionality-driven reduction (e.g., principal components analysis)

^6^ Other data sources beyond the ACS and decennial census included the national longitudinal study of adolescent to adult health (Add Health) survey, the Boston Youth Survey, Summary File 3 Census Data, and the Neighborhood Change Database

^7^ The distribution of measures across levels of geographic analysis is not shown here because many measures in our dataset were adapted and applied to multiple levels of geography

^8^ The census tract of the school the child attends

##### Indicators of nSES

Income was the most often included domain (Table 3, Supplemental Table 1), and 33 different indicators were used to represent this domain. Twenty-one indicators were used to describe education; 12 of which focused on capturing adult education levels in the community, and nine indicators from the child opportunity index which assess children’s educational quality (e.g., number of early child education centers within a five-mile radius, the percent of students in elementary schools eligible for free and reduced lunches, third grade-reading and math proficiency). There were 22 indicators used to capture employment; beyond employment and unemployment rates, some of these indicators explored occupational prestige (e.g., managerial/white collar work versus blue collar or manual labor). Six different indicators were used to assess marital status; most studies that included a marital status indicator attempted to capture single motherhood. There were 30 different indicators in the housing domain. These included eight indicators centered around housing costs and value (e.g., median home value, median rent, or median monthly mortgage). The most used housing quality measure (*n* = 71 studies) was crowding, or the proportion of all occupied housing units with more than one person per room. There were nine indicators in the transportation domain; two focused on vehicle access while the others assessed explored commuting and walkability.

**Table 3:** Summary of the most commonly used types of neighborhood socioeconomic status (nSES) indicators in each domain (*n* = 206 studies)

Domain	Indicator type	Number of indicators
Total number of indicators		**170**
Income		**33**
	Federal poverty limit	11
	Median income	12
	Assistance based	4
	Other income^1^	6
Education		**21**
	Educational attainment	12
	Child opportunity index education indicators^2^	9
Employment		**22**
	Employment status	8
	Occupation	9
	Gender-specific employment	5
Marital status		**6**
	Single parenthood	3
	Other	3
Housing		**30**
	Housing cost/value	8
	Housing quality	12
	Housing age	10
Transportation		**9**
	Vehicle access	2
	Commuting and walkability	7
Other^3^		**49**
	Demographic	16
	Health insurance	3
	Built and physical Environment	13
	Crime	8
	Psychosocial	6
	Miscellaneous^4^	3

Table 3 is a high-level summary of the different indicator types across each extracted nSES domain. A complete list of all 170 extracted indicators can be found in Supplementary Table 1

^1^ Examples of other income indicators include an income disparity index, per capita income, and the percentage of elementary students eligible for free and reduced lunch

^2^ While most education indicators focus on parental education attainment, the child opportunity index education indicators focus on capturing the educational environment in middle childhood (e.g., number of early childhood education centers within a 5-mile radius, percent of three- and four-year-olds enrolled in nursery school, preschool or kindergarten, third-grade reading, and math proficiency)

^3^Indicators captured in this domain are not necessarily traditional SES indicators but were extracted because studies incorporated them into their nSES measures

^4^ Indicators that fell under this category were the number of summer days with maximum temperature above 90F, HIV prevalence and cost of childcare

Forty-eight additional indicators did not fall into one of the a priori domains. Sixteen of those indicators captured demographic information (e.g., percent minority or the number of individuals living with disability). Three were healthcare resource-oriented indicators, such as the percentage of children under six on public insurance. Thirteen measured the built or physical environment (e.g., percent of impenetrable surface areas, the average number of superfund sites within a 2-mile radius, index of toxic chemicals released by industrial facilities, air pollution, and greenspace access) highlight differential environmental exposures. There were eight indicators that measured crime, and an additional six psychosocial indicators, including exposure to violence, and perception of neighborhood physical disorder, while three remained that were uncategorizable (number of summer days with maximum temperature above 90F, HIV prevalence, and cost of childcare).

##### Data Sources

Government public use datasets were the most used data source for nSES indicators. Most studies (96% [*n* = 198]) and measures (89% [*n* = 108]) used either the ACS or the decennial census as a data source. The American Community Survey (ACS) was the primary data source for 59.2% of studies and 38.8% of measures. Thirty-two (19.9%) studies and 29 (23.9%) measures incorporated data from alternative data sources, e.g., study-specific survey data and state assessments to supplement the data derived from national public use datasets (Supplemental Table 2) [33].

##### Neighborhoods

Approximately 80% of studies used a census-based boundary to define neighborhoods, and the most used geographic unit of analysis was census tracts (119 [57.8%] studies). Approximately 24% (*n* = 49) of studies used census block groups, and three used the census tracts of the schools that children attended instead of the census tract of residence. Thirty-four (16.5%) studies used zip codes, and three did not specify the level of geography used but stated that they were neighborhood measures. Four studies used community-defined neighborhood areas [33–36]. Nine articles used area-level measures collected via the ACS as a proxy for individual-level SES rather than to measure nSES. Only 11 articles explicitly classified neighborhoods as rural or urban, and only three articles, Bagley et al. (2018), Mennis et al. (2022), and Tomayko et al. (2016), made special considerations for measurement in rural communities [37, 38•, 39].

#### Other Study Characteristics

Approximately 74% (*n* = 152) of the studies we extracted focused on general pediatric populations, while the rest focused on children with chronic or special conditions (e.g., cancer, cystic fibrosis) (Table 4). Approximately 28% (*n* = 57) of studies evaluated birth and infancy outcomes, 43 (20.9%) included preschool-aged children, and 81 (39.3%) included school-age children. Adolescents were the most frequently studied age group (95 studies (46.1%)).

**Table 4:** Summary of study populations and child health outcomes (*n* = 206 studies)

Population type	No. (%) ^1^
General	152 (73.8%)
Chronic/special conditions	54 (26.2%)
Age (years)	
Perinatal and infancy (0–1)	57 (27.7%)
Early childhood (2–5)	43 (20.9%)
Middle childhood (6–12)	81 (39.3%)
Adolescence (13+)	95 (46.1%)
Not specified	42 (20.4%)
Outcomes	
Asthma or other respiratory	14 (6.8%)
Birth outcomes	24 (11.7%)
Growth and adiposity	24 (11.7%)
Health behaviors	46 (22.3%)
Hospital care/access/treatment	38 (18.4%)
Injury/child abuse/maltreatment	23 (11.2%)
Mental health/neurocognition	40 (19.4%)
Rare/serious/infectious disease^2^	21 (10.2%)
Sleep	9 (4.4%)
Substance use	14 (6.8%)
Other^3^	45 (21.8%)

^1^ Age and outcome sections will not add up to 206, as they were collected as check all that apply

^2^ Due to the relatively low number of studies that focused on cancer as an outcome, cancer was grouped with other rare, serious, and infectious diseases

^3^ Examples of outcomes that fell into the other category include but are not limited to vaccination rates, quality of life assessments, blood lead levels, risk of incarceration, average life expectancy, teen pregnancy, and self-reported life expectancy

There was substantial heterogeneity in the outcomes studied. The three most studied categories were health behaviors, other health outcomes, and mental health/neurocognition. Forty-six (22.3%) studies explored different health behaviors (e.g., physical activity, diet). Approximately 22% (*n* = 45) of studies explored health outcomes that did not fit our 10 pre-defined categories. Examples of health outcomes captured in the other category include blood lead levels, cardiovascular risk factors, and teen pregnancy. Mental health and neurocognition were the most frequently studied outcome with 40 (19.4%) of studies examining it.

## Discussion

We identified 24 established and 97 study-specific nSES measures used in 206 studies of the association between nSES and child health. Within the 121 different nSES measures, we identified 170 indicators spanning seven a priori domains. The variation across age groups and health outcomes in our review highlights the interest in studying the effects of nSES on a myriad of child health outcomes. The definitions of neighborhood and measurement of nSES were primarily centered around census tracts in urban areas, and the effects on rural children remain largely unexplored. We found notable variation in the domains, indicators, and methods used in creating nSES measures, which we interpreted as evidence of a lack of a robust theoretical foundation.

### Measures

This review focused on nSES measure and indicator selection rather than the association between nSES and health outcomes. We chose to focus on measurement because a clear understanding of how nSES is being measured is key to accounting for measurement error, causal inference, and targeted resolution of environmental injustices. The unequal burden of environmental exposures in low SES neighborhoods reflects broader socioeconomic disparities and highlights how accurate measurement of nSES is key to targeting environmental justice efforts in the most vulnerable places. Past reviews have noted the importance of SES in environmental justice research as well as the variability in measuring nSES and defining neighborhoods, but their primary focus was not describing indicators, domains, or measure creation [12, 22, 28]. Van Vuuren et al. noted variation in indicator selection; we build upon those findings by extracting more recent articles, with a greater focus on domain and indicator selection for nSES measurement in the USA [22].

The notable variation in measures, domains, indicators, and neighborhood definitions suggests a lack of theoretical clarity on nSES. Variation itself is not inherently problematic; including different indicators might better align with a specific theoretical framework, hypothesized causal pathway or health outcome, or the study’s research goals. For example, socioeconomic positioning is a *relational* concept of how individuals and groups stand in approximation to each other [3]. In contrast, socioeconomic status is *resource oriented* and refers to the differences between individual groups’ material possessions and ability to possess resources [3]. This would drive indicator selection toward indicators such as median home value. Even within nSES, there are more specific theories, such as social class-based conceptions of nSES. A class-based perspective highlights structural processes that shape access to social and material resources. This might steer indicator selection toward more upstream factors, such as school district funding levels.

Studies that do not report theoretical frameworks or decision-making processes for indicator selection are more open to ambiguity and misinterpretation, which can make it difficult to build from existing work. A frequent example is using racial composition data as a proxy for nSES: 32 studies were excluded from our review because racial composition was the sole measure of nSES, and 99 (48.1%) studies in our review incorporated racial, ethnic, or demographic indicators in their nSES measures. Using racial and ethnic composition as a proxy for nSES fails to address the environmental and structural differences that result in increased adverse exposure to low-income minorities [40]. Racial and ethnic compositions are distinct from nSES, and treating them as proxies in health research can obfuscate fundamental drivers of inequity [40–42]. Structural racism refers to the societal perpetuation and endorsement of discrimination across multiple interconnected systems, including but not limited to policy, housing, education, employment, transportation, criminal justice, healthcare, income, and wealth [43••]. Historical patterns of disinvestment and discriminatory policies due to structural racism have perpetuated inequalities in environmental exposure and access to resources in the USA [44]. Instead, conceptualizing race and ethnicity as distinct but related constructs can allow for a more nuanced multi-level comparison with nSES that allows for an intersectional examination between individual, neighborhood, and broader environmental exposures.

Theory should be used not only to guide nSES indicator selection but also the methodological process of measure creation. It is difficult to capture the complex and multi-dimensional nature of nSES, making it hard to justify single-indicator measures [29••]. Nevertheless, we excluded 143 studies during the full-text review because nSES was measured using a single indicator. Furthermore, none of the 143 studies we excluded provided a theoretical justification as to why nSES could be measured using a single indicator. The nSES measures in our review were created using a variety of methods. The majority of nSES measures were indices (63%) followed by measures generated via principal components analysis (26%) and latent variable (16%) methods. Very few papers we extracted discussed the selection of a statistical approach at length. While all these methods can be used to select a smaller set of relevant indicators and create a composite measure of nSES, each has a distinct theoretical foundation and assumptions about the relationship between the indicators and the indicators and final measure. The selection of a method should be based on the goals of the study and the desired result (e.g., index, scale), and a brief discussion of theoretical rationale behind the choice to use a particular analytical method might enhance an audience’s understanding of the findings and for future research to build off a more solid foundation.

### Neighborhood Definition

We found data aggregation and geographic unit trends similar to those reported by Arcaya et al. [28]: Census Bureau was the most common data source, and census tracts were the most common geographic unit. This suggests a lack of growth in developing new methods to define sociologically relevant neighborhood boundaries despite many researchers cataloging the shortcomings of administrative units for neighborhood health research [45].

Defining and selecting an appropriate geographic boundary that aligns with the studied area poses theoretical and practical challenges. Researchers must often make do with the best available data, which frequently means using administrative units as proxies for neighborhoods because they are readily available and can be standardized nationally [21••]. However, administrative proxies do not capture the relevant geography as it is often conceptualized in neighborhood health research, where neighborhoods are socio-physical communities with clear boundaries known to their residents [46]. The scale and shape of geographic aggregation can influence data measurement and interpretation [47, 48]. Thus, it is vital for studies to explain the level of geographic specificity used because this changes the interpretation of nSES [27].

An example of how geographic units can influence the interpretation of nSES can be seen through the contrasting results of two studies that used the same level of geography and nSES measure. Bagley et al. examined nSES effects on children’s sleep in rural and semi-rural Alabama [37]. Bagley et al. adapted a nSES measure from Pabayo et al. that reported a significant association between nSES and children’s sleep outcomes in Boston [37, 49]. Both studies used census tracts as a neighborhood proxy. However, while Pabayo et al. [49] found significant associations between nSES and children’s sleep, Bagley et al. [37] found no significant associations were detected in Alabama. It is difficult to ascertain whether this is because there was no nSES effect in this study population, or because the “neighborhood” measurement and nSES interpretation differ between Boston and Alabama due to the variation in size of census tracts between urban and rural areas. Census tracts vary in geographic size based on population; they typically represent a population size between 1200 and 8000 people [50]. In urban areas, standardized administrative units like census tracts align more closely with functional definitions of neighborhood, allowing for a reasonable comparison between urban children across the country.

In rural and semi-rural areas, census tracts may not be a valid proxy of a neighborhood because the census tract area is much larger to encompass the required population size [51]. This makes it challenging to study the nSES effect on children in non-urban areas and increases the likelihood of measurement error. To address this issue, Mennis et al. [38] used census block variables to create one-mile Euclidean buffers around each child’s home, using population-weighted nSES data based on the proportion of the population of the block group(s) in the buffer zone. This allowed Mennis et al. to create a more specific measure of nSES within each participant’s buffer. This method increased comparability and reduced measurement error by standardizing the geographic boundaries of neighborhoods between urban and rural communities [38]. However, defining a neighborhood this way does not account for administrative or political boundaries, roads, access to goods and services, or residents’ perceptions of neighborhood boundaries.

### Limitations

The lack of uniformity in how studies are indexed made it more challenging to conduct a comprehensive review. We had to rely on overly broad MeSH terms like “socioeconomic factors,” which returned more than a half-million results, and “residence characteristics” (> 80,000 papers). Given our goal of analyzing nSES measure selection and creation, small changes in the search procedure or extraction protocol would likely produce similar findings. Additionally, we did not critically appraise the individual quality of the nSES measures extracted or include any grey literature on the subject. Although we used well-defined selection criteria to limit the number of articles defined and followed the PRISMA extension for scoping reviews, it is possible that this could have impacted our results.

## Recommendations for nSES in Environmental Health Research

### Measures

We recognize that while measurement standardization is often the goal in epidemiology, it is not always practical or feasible to achieve this, as researchers often must make do with the data available. We offer up the following recommendations to guide consideration of how indicator, geographic unit, data source selection can affect nSES measurement and interpretation. We recommend the following guidelines adapted from Oakes and Kaufman for the measurement of SES [52]. These include 1) start with a clear hypothesis of the pathways (e.g., material, psychosocial, relational) between nSES and the specific child health outcome under study; and 2) select nSES domains and indicators that are most salient in your theoretical framework and study population. In an environmental justice framework, the effects of nSES on child health, age, and developmental stage are important facets of the study population to consider. Early childhood is a critical period, and access to material resources (e.g., access to healthcare, childcare, housing quality) can impede or promote developmental potential [53]. Psychosocial indicators (e.g., crime rate, social fragmentation) might be more useful when studying health behaviors in adolescents who are independently moving through their neighborhood environment [54]. When using existing nSES measures, some indicator selections within a domain of interest may need to be modified for the study population. For example, the COI uses grade-level assessments as an indicator of education, which might give us greater insight into child nSES in a way that traditional nSES education indicators, like parental educational attainment, may fail to capture [55].

There are many measures of nSES or deprivation that may meet the needs of a given study. The advantages of existing measures include ease of application and comparison to other studies. Creation of a new measure is a significant undertaking and may not be necessary, despite being a common approach in the studies in this review. However, there may not be a measure that meets the goals of the study or that is appropriate for the population under study. Supplementary Table 3 is a comprehensive catalog that can be used to compare existing nSES measures used in the last decade in child health research. Additionally, nSES is a time-varying measure, and new measures may need to be developed as new indicators become relevant. For example, broadband internet access is an emergent indicator, while indicators such as female-headed households may reflect outdated theories of nSES. Other indicators that may represent climate vulnerability, like proximity to flood plains or wildfire risk, might become more relevant indicators of nSES as the climate crisis continues.

### Neighborhood

Selecting the level of geography that is both a relevant representation of a neighborhood for the study population and has available data continues to be a challenge. Locally defined “named neighborhoods” remain the gold standard for measurement and the intended target of most of the neighborhood effects research but can only be used in specific settings and may not align with key data sources. Younger children may access smaller geographic areas in their daily activities than older children, altering the desirable neighborhood unit for a specific study. More methodological development is needed to understand and delineate “neighborhood” contexts in non-urban areas. A Euclidean radius around a child’s home may allow for greater standardization across different contexts but may not fully capture neighborhoods as recognized by their inhabitants.

An additional consideration for the selection of a geographic unit, largely missing from the studies in this review, is the potential for intervention. Selecting a geography based on its potential for intervention can allow not only for the identification of environmental hazards but also provide the opportunity to improve access to greenspaces, change zoning laws to improve environmental exposure, pass housing quality standards, or mediate the development of transportation infrastructure. Larger administrative units such as towns, cities, and counties have policy and budget-making authority that make these units more amenable to interventions than smaller units such as census tracts or distance-based buffers. Named neighborhoods fall somewhere in between, as they are unlikely to wield policy-making power but can have strength as organized political units via neighborhood associations.

## Conclusion

All manuscripts should describe theoretical assumptions, definitions, and decision-making processes that the study team underwent to select an established nSES measure, modify an existing measure, or build a new measure of nSES. This is especially important for studies that take an environmental justice framework as an environmental justice perspective takes into consideration many composite measures and disentangling them allows for more precise causal inference. More specific and standardized MeSH terms and keywords associated with nSES are needed to make it easier for others to access newer methodologies, theories, and studies to continue to expand knowledge in this important area of research. This review shows that there are several studies of the association between nSES and children’s health; however, incorporating theory into measure selection and/or creation, clearly stating the study’s definition and interpretation of both nSES and neighborhood, will create a clearer understanding of the study objectives and results. Rigorous and well-reported measurements are critical for evaluating and ultimately addressing health disparities at the neighborhood level.

### Supplementary Information

ESM 1 (DOCX 74.8 kb)

ESM 2 (DOCX 42 kb)

## Data Availability

A covidence extraction file and an endnote file containing all extracted articles included in the manuscript can be made available upon request.
