# Supplementary material for: Unpacking Neighborhood Socioeconomic Status in Children’s Health Research from an Environmental Justice Perspective: A Scoping Review
Source: Curr Environ Health Rep. 2024 Apr 10;11(2):288–99. doi: 10.1007/s40572-024-00445-8 (PMC11081999; doi:10.1007/s40572-024-00445-8)
Supplement: Supplementary file 1 — (DOCX 74.8 kb) [file 40572_2024_445_MOESM1_ESM.docx]

| **Supplementary Table 1: A comprehensive list of all indicators used by nSES domain (n= 206 studies^1^)** | | | |
| --- | --- | --- | --- |
| **Total number of indicators across all domains n = 170** | | | |
| **Domain (N indicators)** | **Indicator Type**  **(N)** | **Indicator** | **Frequency (N Studies)** |
| **Income (33)** |  |  | **202** |
|  | **Federal Poverty limit (11)** |  |  |
|  |  | Percent living below the federal poverty line | 117 |
|  |  | Percent of population below 150% of the federal poverty line | 53 |
|  |  | Percentage of households with high income | 2 |
|  |  | Percent of households above the poverty line | 1 |
|  |  | Percent of the population <18 living in poverty | 1 |
|  |  | Percentage of adults >65 living below the federal poverty line | 1 |
|  |  | Zip Code Tabulated Areas with >20% of households living below the federal poverty line | 1 |
|  |  | Percent of households below 200% federal poverty line | 1 |
|  |  | Tract poverty rate | 1 |
|  |  | Black poverty rate | 1 |
|  |  | Percent of households with income above state median | 1 |
|  | **Income (12)** |  |  |
|  |  | Median household income | 116^2^ |
|  |  | Percent of households with interest or dividends or net rental income | 10 |
|  |  | Proportion of residents with income other than wages/salary | 1 |
|  |  | Median income ratio as a percentage of the state’s median income | 3 |
|  |  | Percent of households earning less than $15,000 | 4 |
|  |  | Percent of households earning less than $25,000 | 3 |
|  |  | Median annual income less than $30,000 | 3 |
|  |  | Percent of families with incomes between $25-49,000 | 1 |
|  |  | Percent of families with incomes greater than $50,000 | 2 |
|  |  | Percent of household earning over $75,000 | 8 |
|  |  | Percent of households with earning more than $100,000 | 3 |
|  |  | Per Capita income | 6 |
|  | **Public Assistance (4)** |  |  |
|  |  | Percent of households receiving public assistance | 53 |
|  |  | Percent of households receiving SNAP or food stamps | 7 |
|  |  | Percent of elementary students eligible for free and reduced lunch | 5 |
|  |  | Percent of residents who receive supplemental social security income | 2 |
|  | **Other Income (6)** |  |  |
|  |  | Income Disparity | 52 |
|  |  | Gini Index Income Inequality | 6 |
|  |  | Family affluence^3^ | 2 |
|  |  | Family income to needs ratio^4^ | 2 |
|  |  | Minimum income for each MSA required to maintain a "sage and decent standard of living"^5^ | 1 |
|  |  | Black income inequality | 1 |
| **Education (21)** |  |  | **174** |
|  | **Educational Attainment (12)** |  |  |
|  |  | Percent of population >25 with at least than a high school education | 74 |
|  |  | Percent of the block groups population aged > 25 years with < 9 years of education | 53 |
|  |  | Percent of adults>24 years who are college educated | 51 |
|  |  | Percent of people without a high school diploma/percent of the population with less than a HS education | 41 |
|  |  | Educational level | 4 |
|  |  | Proportion of persons >25 without a bachelor's degree | 2 |
|  |  | Percent of females with a bachelor's degree or more | 1 |
|  |  | High School dropout rate | 1 |
|  |  | Proportion of individuals aged 25 or older who have completed 16 or more years of education | 1 |
|  |  | Maternal education (proportion greater than 10%) | 1 |
|  |  | Low education (if the tract proportion of residents with less than a HS education was greater than the grand mean) | 1 |
|  |  | High education (if the tract proportion of residents with a college degree was higher than the grand mean) | 1 |
|  | **Child Opportunity Index Education Measures^6^ (9)** |  |  |
|  |  | Number of early childhood education centers within a 5-mile radius | 5 |
|  |  | Number of high quality (NAEYC) accredited centers within a 5-mile radius | 5 |
|  |  | Percent of 3- and 4-year-olds enrolled in nursery school, preschool, or kindergarten | 5 |
|  |  | Third grade reading proficiency | 5 |
|  |  | Third grade math proficiency | 5 |
|  |  | High school graduation rate (percent of ninth graders graduating on time) | 5 |
|  |  | Advanced (AP) course enrollment | 5 |
|  |  | Percent of 18–24-year-olds enrolled in college within a 25-mile radius | 5 |
|  |  | Percent of teachers in a school district in their first or second year | 5 |
| **Employment (22)** |  |  | **172** |
|  | **Employment (8)** |  |  |
|  |  | Percent unemployed | 122 |
|  |  | Percent employed | 5 |
|  |  | Percent change in employment | 3 |
|  |  | Percent of 16–19-year-olds who were not in school, labor force, or military and have not completed high school or GED equivalent | 2 |
|  |  | Percent of employed people in service or production occupations | 2 |
|  |  | Percent of working residents who are in manufacturing industries | 1 |
|  |  | Percent with blue collar jobs | 1 |
|  |  | Percent professional vs construction/lap or other occupations | 1 |
|  | **Occupation (9)** |  |  |
|  |  | Percent white collar employment | 54 |
|  |  | Percent of adults in managerial/professional jobs | 29 |
|  |  | Percent of population in working-class jobs | 2 |
|  |  | Percent of employed people in service or production occupations | 2 |
|  |  | Percent of working residents who are in manufacturing industries | 1 |
|  |  | Percent with blue collar jobs | 1 |
|  |  | Percent professional vs construction/lap or other occupations | 1 |
|  |  | Percent of people employed in high working-class occupations (defined by the study as single variable that combined the following ACS occupation groups: managerial, business, science, and arts occupations) | 1 |
|  |  | Percent of people employed in material extraction jobs (e.g., mining or timber harvesting) | 1 |
|  | **Gender-Specific Employment (5)** |  |  |
|  |  | Male unemployment rate | 11 |
|  |  | Female employment rate | 3 |
|  |  | Female unemployment rate | 2 |
|  |  | Percent males in professional occupations | 1 |
|  |  | Percent of mothers of young children that are unemployed | 1 |
| **Marital Status (6)** |  |  | **131** |
|  | **Single Parenthood (3)** |  |  |
|  |  | Percent of single parent homes | 76 |
|  |  | Percent of female headed households | 35 |
|  |  | Percent single-mother households | 7 |
|  | **Other (3)** |  |  |
|  |  | Percent of people older than 15 who are married | 1 |
|  |  | Percent of married couple families with their own children | 1 |
|  |  | Percent of non-intact families with children | 1 |
| **Housing (30)** |  |  | **141** |
|  | **Cost/Value (8)** |  |  |
|  |  | Median home value | 73^7^ |
|  |  | Percent owner-occupied households | 67 |
|  |  | Housing cost/Median rent | 57 |
|  |  | Median monthly mortgage | 53 |
|  |  | Median value of owner-occupied housing units | 3 |
|  |  | Percent renter or owner costs in excess of 50% of income | 2 |
|  |  | Median home value (less than $100,000) | 1 |
|  |  | Upper quartile value of owner-occupied housing units in the tract | 1 |
|  | **Housing Quality (12)** |  |  |
|  |  | Crowding (proportion of all occupied housing units with more than 1 person per room) | 71 |
|  |  | Number of households without a telephone | 52 |
|  |  | Percent of vacant housing units | 16 |
|  |  | Percent renter occupied housing | 11 |
|  |  | Percentage of persons living in group quarters | 9 |
|  |  | Mobile homes | 9 |
|  |  | Proportion of living in multi-unit structures | 8 |
|  |  | Number of total homes | 1 |
|  |  | Number of homes needing major repairs | 1 |
|  |  | Number of homes with and without refrigerators, radios, and heating | 1 |
|  |  | Percent of households with internet access | 1 |
|  |  | Vox score (proportion of lead exposed homes, housing age) | 1 |
|  | **Housing Age (10)** |  |  |
|  |  | Percent of housing units built in 1939 or earlier | 2 |
|  |  | Percent of housing units built from 1940-1949 | 2 |
|  |  | The share of housing units built before 1940 | 3 |
|  |  | The share of housing units built before 1950 | 2 |
|  |  | The share of housing built before 1980(proportion greater than 50%) | 1 |
|  |  | Percent living in the same house since 1985 | 1 |
|  |  | Percent of residents in the same home since 1995 | 2 |
|  |  | Age of owner-occupied Units | 1 |
|  |  | Median house age (as a proxy for sub-urbanicity) | 1 |
|  |  | Proportion of residents who have lived in the same house for less than 5 years | 1 |
| **Transportation (9)** |  |  | **93** |
|  | **Vehicle Access (2)** |  |  |
|  |  | Percent of households with vehicles | 65 |
|  |  | Percent of households without a car | 3 |
|  | **Commuting & Walkability (7)** |  |  |
|  |  | Percent of workers commuting more than one-hour one way | 5 |
|  |  | EPA walkability Index | 5 |
|  |  | Child walkability^8^ | 1 |
|  |  | Percentile of count of vehicles at major roads per meter within 500 m as compared to the average in the US | 1 |
|  |  | Frequency of transit service per hour within 0.25 miles | 1 |
|  |  | The fraction of workers who commute less than 15 minutes | 1 |
|  |  | Cyclomatic index (the number of route alternatives between intersections around a respondent home in order to assess accessibility) | 1 |
| **Other (49)** |  |  | **128** |
|  | **Demographic (16)** |  |  |
|  |  | Percent African-American | 15 |
|  |  | Percent minority | 15 |
|  |  | Percent of population older than 5 who speak English less than well | 12 |
|  |  | Percent of people younger than 18 or older than 64 | 10 |
|  |  | Percent foreign born | 8 |
|  |  | Ethnic heterogeneity^9^ | 8 |
|  |  | Number of individuals older than 5 living with disability | 8 |
|  |  | Percent Hispanic or Latino | 6 |
|  |  | Proportion speaking a language other than English at home | 3 |
|  |  | Percent of residents 65 and older | 3 |
|  |  | Percent of residents under the age of 18 | 2 |
|  |  | Proportion of households where no one >14 speaks English well or English only | 2 |
|  |  | Immigrant concentration | 1 |
|  |  | Racial segregation | 1 |
|  |  | Combined neighborhood proportion of Asian-Americans and Pacific Islanders | 1 |
|  |  | Proportion of White residents | 1 |
|  | **Health Insurance (3)** |  |  |
|  |  | Percent of Children under the age of 6 on public insurance | 6 |
|  |  | Percent of the population without health insurance | 1 |
|  |  | Cost of healthcare | 1 |
|  | **Built and Physical Environment (13)** |  |  |
|  |  | Urban Rural classification (RUCA) | 5 |
|  |  | Proportion of the tract that is urban | 3 |
|  |  | Tract population density | 3 |
|  |  | Percent of impenetrable surface areas such as rooftops, roads, or parking lots | 5 |
|  |  | Average number of Superfund sites within a 2-mile radius | 5 |
|  |  | Index of toxic chemicals released by industrial facilities | 5 |
|  |  | Mean estimated microparticle (PM 2.5) concentration | 5 |
|  |  | Mean estimated 8-hour average ozone concentration | 5 |
|  |  | Normalized Vegetation Difference Index (Greenspace Access) | 1 |
|  |  | Perceptions of park access | 1 |
|  |  | Change in the number of business establishments | 1 |
|  |  | Tract level-built environment variables^10^ | 1 |
|  |  | Alcohol outlet density^11^ | 1 |
|  | **Crime (8)** |  |  |
|  |  | Crime rate | 5 |
|  |  | Number of juvenile arrests for violent crimes and serious nonviolent crimes | 2 |
|  |  | Neighborhood crime index^12^ | 2 |
|  |  | Community violence (number of deaths to children <17 due to firearms) | 1 |
|  |  | Number of drug-related juvenile arrests | 1 |
|  |  | Number of adult arrests | 1 |
|  |  | Number of adult violent and nonviolent offenses | 1 |
|  |  | Index of rate of criminal assaults | 1 |
|  | **Psychosocial (6)** |  |  |
|  |  | Social fragmentation | 2 |
|  |  | Perception of neighborhood safety | 1 |
|  |  | Exposure to violence | 1 |
|  |  | Perception of neighborhood physical disorder | 1 |
|  |  | Measure of community norms unfavorable to drug use | 1 |
|  |  | Maternal Sensitivity | 1 |
|  | **Miscellaneous (3)** |  |  |
|  |  | Number of summer days with maximum temperature above 90F | 5 |
|  |  | HIV prevalence | 1 |
|  |  | Cost of childcare | 1 |

We extracted all indicators that were included in nSES measures regardless of whether they fit into an nSES domain, which is why there are 48 indicators captured in the other domain, some of which do not appear to measure nSES.

^1^ There are 206 studies that use 121 measures of nSES, and 170 indicators across all 7 domains (income, education, employment, marital status, transportation, and other)

^2^ Median household income is reported as the cumulative total of studies that used median household income (n = 113) and log median household income (n=3).

^3^Health-behavior in school-aged children (HBSC) family affluence scale is a summed score of four-items: family car ownership (0, 1, 2+) and computer (0, 1, 2+) ownership, the past-year frequency of family vacations (0, 1, 2+) and whether respondents had their own bedrooms incorporated alongside other variables to construct an nSES measure [1].

^4^ Family income to needs ratio is calculated by dividing the total family income by the U.S. census poverty threshold for the family size for each year.

^5^ The Economic Policy Institutes family budget calculator which estimates community specific costs for ten family types (one or two adults with zero to four children).

^6^ While most education indicators focus on parental education attainment. The Child Opportunity Index education indicators focus on capturing the educational environment in middle childhood.

^7^ Of the 73 studies that looked at median home value three used the log median home value.

^8^ One-half mile represented a 10-min walk for a student. The average distances between the students’ homes to the nearest grocery store, convenience store, fast food restaurant, and park were calculated using ArcGIS to assess students’ neighborhood walkability [2].

^9^Ethnic heterogeneity is an index calculated by summing the squared proportions of each racial/ethnic group in the study population and subtracting that from one, with a higher number indicating a higher level of ethnic heterogeneity.

^10^ The total number of grocery stores, convenience stores, fast food restaurants, and parks within each tract and 20-m buffer [2].

^11^ Alcohol outlet density was measured by the number of liquor, beer, and wine stores within a 5km radius of participants homes, and then categorized into quartiles [3].

^12^ Indices of property (burglary, motor vehicle theft) and personal crime (murder, rape, and assault) were standardized at the census block level and weighted equally within each crime category and then categorized into quartiles.

| **Supplementary Table 2: Alternative Data Sources to the American Community Survey and the Decennial Census for nSES Indicators** | | |
| --- | --- | --- |
|  | **N Papers (n= 206)** | **N Measures (n=121)** |
| American Community Survey | 122 | 47 |
| Decennial Census | 76 | 61 |
| **Alternative Data Sources** | **32** | **29** |
| The National Longitudinal Study of Adolescent to Adult Health (Add Health) Survey | 7 | 5 |
| Unspecified U.S. Census Bureau Data | 3 | 3 |
| Boston Youth Survey | 2 | 2 |
| Summary File 3 Census data^1^ | 2 | 2 |
| Neighborhood Change Database | 2 | 1 |
| US Federal Bureau of Investigation Uniform Crime Reporting Program | 1 | 1 |
| Opportunity Atlas | 1 | 1 |
| Economic Policy Institute Basic Family Budget | 1 | 1 |
| Project on Human Development in Chicago Neighborhoods | 1 | 1 |
| Waves 2-5 of the 1997 National Longitudinal Survey of Youth | 1 | 1 |
| North Carolina State Center of Health Statistics | 1 | 1 |
| Baltimore City Community Statistical Areas and Police Records | 1 | 1 |
| Health Care Cost Institute | 1 | 1 |
| National Historical Geographic Information System | 1 | 1 |
| The Atherosclerosis Risk in Communities (ARIC) Study | 1 | 1 |
| Census Bureau Business Pattern | 1 | 1 |
| Crime Open Database | 1 | 1 |
| Primary Land Use and Tax Lot Output Data Files | 1 | 1 |
| NYC Department of Health and Mental Hygiene Community Health Survey | 1 | 1 |
| Maryland All Payer 2013-2016 Dataset | 1 | 1 |
| Adolescent Brain Cognitive Development (ABCD) Study | 1 | 1 |

Approximately 96% (n = 198) of all studies and 88.5% of measures (n =108) used either the American Community Survey (ACS) or the

Decennial Census as a data source when creating nSES measures. This table is a list of the 21 alternative data sources used by 32 different studies.

^1^ Summary File 3 is a census derived dataset from 2002 that contains sample data compiled from the questions asked of a sample of all people and housing units. It pre-dates the ACS, which launched in January of 2005, and contains similar, but truncated data to the ACS.

| **Supplementary Table 3: A List of Established nSES Measures** | | |
| --- | --- | --- |
| **Measure Name** | **Citation** | **Description of Index** |
| **Area Deprivation Index (ADI)** | (Kind & Buckingham, 2018) [4] | The ADI was originally created by the Health Resources and Services Administration (HRSA) more than three decades ago. Since then, Amy Kind and her team at the University of Wisconsin-Madison have refined, adapted, and validated it at the census block group level to be a composite measure of 17 census derived indicators across six domains: income, education, employment, marital status, housing, and transportation. The ADI scores for each census block group are publicly available through the Neighborhood Atlas Project. |
| **Neighborhood Deprivation Index (NDI)** | (Messer et al., 2006) [5] | Based on the findings of a literature review, Messer et al., (2006) developed a composite measure at the census tract level. It incorporates twenty census derived indicators across five domains: income, education, employment, housing, residential stability. It also includes two racial composition variables. |
| **Social Vulnerability Index (SVI)** | (Centers for Disease Control and Prevention/Agency for Toxic Substances and Disease Registry/Geospatial Research, 2020) [6] | Developed by the CDC/ASTDR to assess communities that might be at greater risk during natural, human-caused disasters, or disease outbreaks. SVI is a census tract measure that uses 16 census-derived indicators across five domains: income, education, employment, marital status, transportation. It also includes variables of the proportion of people over the age of 65 or under 17, individuals who live with disability, minority status, and the ability to speak English. |
| **Child Opportunity Index (COI)** | (Child Opportunity Index 2.0, 2023) [7] | Developed at Brandeis as a part of the Diversity Data Kids project. It is a composite measure based on 29 indicators across six domains: income, education, employment, marital status, housing, and transportation. It also includes additional variables that look at environmental variables such as airborne microparticles, ozone concentration, extreme heat exposure, and hazardous waste dump sites. There are also indicators of access to healthy food, greenspace, and health insurance coverage. It is available at the census tract level and the zip code level. |
| **Diez-Roux Deprivation Score** | (Diez Roux & Mair, 2010) [8] | The National Cancer Institutes created an index using 13 indicators across four domains (income, education, occupation, and housing conditions) based off of Diez-Roux & Mair’s (2010) findings that show that features of neighborhoods can contribute to health inequalities. Diez-Roux & Mair did not formally create an nSES measure but tested twenty different indicators for significant associations on health, but many people reference adapting the significant indicators into an index or a score as the “Diez-Roux Deprivation Score”. |
| **Brokamp Neighborhood Socioeconomic Deprivation Index** | (Brokamp et al., 2019) [9] | Census tract level measure using six indicators across three domains (income, education and housing) used to measure material deprivation. It also includes an additional indicator of health insurance coverage. The index weights were determined using principal components analysis. The index is available on GitHub (https://github.com/geomarker-io/dep_index) and can be reproduced nationally at both the census tract and zip code level. |
| **Wheeler Neighborhood Risk Score** | (Wheeler et al., 2019) [10] | Census tract level measure using 14 indicators across six domains (income, education, employment, marital status, and other) developed to assess amplified risk of elevated blood lead levels due to lower nSES. |
| **Yost Index** | (Yost et al., 2001) [11] | Census tract level measure with seven indicators across four domains (income, education, employment, and housing). The Yost index has been promoted by the National Cancer Institute and is primarily used in studying nSES effects on cancer outcomes. It is a more streamlined index when compared to the ADI. The Yost index uses seven indicators while the ADI uses 17(Boscoe et al., 2021). |
| **Index of Neighborhood Disadvantage** | (Ross & Mirowsky, 2001) [12] | An index measure created by subtracting the sum of the percent of mother-headed households and the percent living below the poverty line (two indicators of neighborhood disadvantage) from the sum of the percent of adults >24 years who are college educated and the percent of households in the census tract that were owner occupied (2 indicators of neighborhood advantage. Validated at the census tract level. |
| **Cost-of Living Index** | ("Family Budget Calculator,"nd) [13] | The economic policy institute calculates an estimate of the minimum total cost of necessary family expenses (e.g., housing, health care, childcare, transportation, healthcare, other necessities and taxes) at the metropolitan statistical area, county or state level. The measure captures the minimum range for each geography required to maintain a “safe and decent standard of living.” |
| **Distressed Neighborhoods** | (O'Hare & Mather, 2003) [14] | Census tract measure based on the 2000 census but can be used at the zip code level as well. It identifies individuals as living in a distressed community if the score meets three out of the following four criteria. 1) >25.1% of individuals in poverty; 2) >20.7% high school dropout rate; 3) 34.4% female head of households; 4) >27.1% male unemployment rate. |
| **Neighborhood Effects Method** | (Leventhal & Brooks-Gunn, 2000) [15] | Similar to the Diez-Roux score, Leventhal and Brooks-Gunn did not create an nSES measure, but this article is a comprehensive review of the effects of neighborhoods on child and adolescent well-being. The findings from this review and highlight indicators of significance have led to many people referencing the use of the “neighborhood effects method” in developing nSES measures. |
| **Neighborhood Concentrated Disadvantage** | (Molnar et al., 2004) [16] | Based on principal components factor analysis of census tract data. This measure uses three indicators (percentage of residents living below the poverty line, receiving public assistance, and unemployment rate) across two domains (income, and employment). |
| **Modified Darden-Kamel Composite Socioeconomic Index to Measure Socioeconomic Position** | (Darden et al., 2010) [17] | First developed to test socioeconomic differences between white and black communities in Detroit. It is a census tract level measure. The measure uses nine indicators across five domains (income, education, employment, transportation, and housing). We found that it was also used as a proxy for individual level measures of SEP (Moody HA et al., 2016). |
| **Agency for Healthcare Research & Quality SES Index** | (Bonito et al., 2008) [18] | This measure is used at both the block group and tract level. The Index follows the following weighting and indicators.  SES Index Score = 50 + (−0.07*crowded) + (0.08*prop100) + (−0.10*pct_poverty) + (0.11*hhinc100) + (0.10*high_educ) + (−0.11*low_ educ) + (−0.08*pct_unemp).  The index components consisted of the following: percentage of households containing ≥ 1 person per room (crowded); the median home value standardized to range from 0% to 100% (prop100); the percentage of persons below the federally defined poverty level (pct_poverty); the median household income standardized to 0–100 (hhinc100); the percentage of persons age ≥ 25 years with at least 4 years of college (high_educ); the percentage of persons age ≥ 25 years with less than a 12th grade education (low_educ); and the percentage of persons age ≥ 16 years in the labor force who are unemployed and actively seeking work (pct_unemp). |
| **Neighborhood Socioeconomic Position Index** | (Lupo et al., 2015) [19] | Census tract level measure that attempts to capture neighborhood SEP rather than resource deprivation. It uses six indicators across four domains (income, education, employment, and housing. The six indicators are proportion of households below the poverty line, proportion of the population without a high school diploma or equivalent, the proportion of the population unemployed, the proportion of the employed civilian population aged at least 16 years employed in a service or production occupation, proportion of occupied housing units that are renter occupied, and crowding. |
| **Neighborhood SES Index** | (Anderson et al., 2014)  [20] | Census block group level measure. Anderson et al. created different sub-measures for each developmental period citing that the inclusion of certain indicators was more or less applicable based on the developmental period. They used confirmatory factor analysis of the nSES indicators for each developmental period early childhood, middle childhood, and early adolescence. The measure is composed of five indicators across four domains: income, education, employment, marital status. |
| **Neighborhood Risk Index** | (Coley et al., 2016) [21] | Census-tract level measure. Principal component analysis was used to develop this index. The index includes the following census-tract variables: median household income, poverty proportion, unemployment rate, percentage of people 25 years old and older with a high school diploma or more, percentage of households headed by single females with children younger than age 18, percentage of households that received public assistance, the Gini inequality index (a standardized measurement of inequality within census-tracts), and percentage of households residing in rental housing. |
| **Neighborhood Adversity Index** | (Messer et al., 2006) [5] | Messer et al. created a standardized nSES measure based on the findings of a systematic review of existing neighborhood measures. They used principal components analysis (PCA) and exploratory factor analysis to determine which indicators to include. They started with 20 census indicators and ultimately chose to only include eight in their final index across five domains: income, education, employment, marital status, and housing. PCA determined the final weighting of the index. |
| **California Healthy Places Index** | ("California Healthy Places Index,"nd) [22] | Developed by the Public Health Alliance of Southern California, it uses Axis Maps to explore the community conditions that impact life expectancy. The HPI combines 25 community characteristics, like access to healthcare, housing, education, and more, into a single HPI score. |
| **Hardship Index** | (Cohen et al., 2016) [23] | The hardship index is calculated for Chicago community areas. It is a composite area level measure based on six indicators: the unemployment rate of persons aged older than 16 years, per capita income, the percent of residents aged younger than 18 years or older than 64 years, the percent of residents living in crowded housing, the percent of households living below poverty, and the percent of residents over the age of 25 who had not earned a high school diploma. It does incorporate any data on racial segregation. It is combined using the following formula:  X_i_ = ((Y_i_ – Y_min_)/ (Y_max_ – Y_min_)) *100  X and Y represent the standardized and unstandardized components, while Ymin and Ymax denote the minimum and maximum values of those measures. The hardship index score is a composite average of each of the standardized components with scores between 1 and 100, where larger values represent greater hardship. |
| **Yang et al. Index** | (Yang et al., 2014) [24] | Adapted from the Yost index which was created for the decennial census. Yang et al., (2014) recreated the Yost index with ACS data, and then proceeded to exclude indicators that had a coefficient of variation that was greater than 40%. In the end the index uses seven indicators across four domains: income, education, employment, and housing. The measure was developed using principal components analysis to determine the weighting for the index. The measure has been validated at the census block group and tract levels. |
| **Economic Innovation Groups: Distressed Communities Index** | (Kesler, nd) [25] | Developed by the Economic Innovation group, the DCI is a zip code level measure that uses Census Bureau Business Patterns and ACS data. It incorporates seven indicators across four domains: income, education, employment, and housing. It also includes two additional indicators: the percent change in the number of jobs and the percent change in the number of business establishments. |
| **Economic Hardship Index** | (Montiel et al., 2004) [26] | The economic hardship index is calculated in the same way as the hardship index (Chicago) but uses census data to create a measure at the census block level. It is a composite area level measure based on six indicators: the unemployment rate of persons aged older than 16 years, per capita income, the percent of residents aged younger than 18 years or older than 64 years, the percent of residents living in crowded housing, the percent of households living below poverty, and the percent of residents over the age of 25 who had not earned a high school diploma. It does incorporate any data on racial segregation. It is combined using the following formula:  X_i_ = ((Y_i_ – Y_min_)/ (Y_max_ – Y_min_)) *100  X and Y represent the standardized and unstandardized components, while Ymin and Ymax denote the minimum and maximum values of those measures. The hardship index score is a composite average of each of the standardized components with scores between 1 and 100, where larger values represent greater hardship. |

**References:**

1. Currie C, Molcho M, Boyce W, Holstein B, Torsheim T, Richter M. Researching health inequalities in adolescents: the development of the Health Behaviour in School-Aged Children (HBSC) family affluence scale. Social Science & Medicine (1982). 2008;66(6):1429-36. doi: 10.1016/j.socscimed.2007.11.024.

2. Carroll-Scott A, Gilstad-Hayden K, Rosenthal L, Peters SM, McCaslin C, Joyce R, et al. Disentangling neighborhood contextual associations with child body mass index, diet, and physical activity: the role of built, socioeconomic, and social environments. Soc Sci Med. 2013;95:106-14. doi: 10.1016/j.socscimed.2013.04.003.

3. Fairman BJ, Goldstein RB, Simons-Morton BG, Haynie DL, Liu D, Hingson RW, et al. Neighbourhood context and binge drinking from adolescence into early adulthood in a US national cohort. Int J Epidemiol. 2020;49(1):103-12. doi: 10.1093/ije/dyz133.

4. Kind AJH, Buckingham WR. Making Neighborhood-Disadvantage Metrics Accessible - The Neighborhood Atlas. N Engl J Med. 2018;378(26):2456-8. doi: 10.1056/NEJMp1802313.

5. Messer LC, Laraia BA, Kaufman JS, Eyster J, Holzman C, Culhane J, et al. The Development of a Standardized Neighborhood Deprivation Index. J Urban Health. 2006;83(6):1041-62. doi: 10.1007/s11524-006-9094-x.

6. Centers for Disease Control and Prevention/Agency for Toxic Substances and Disease Registry/Geospatial Research AaSP. Social Vulnerability Index Database. In: CDC/ASTDR, editor. https://www.atsdr.cdc.gov/placeandhealth/svi/data_documentation_download.html2020.

7. Child Opportunity Index 2.0. In: Institute for Child YaFP, Heller School for Social Policy and Management, Brandeis University, editor. Waltham, MA: diversitydatakids.org; 2023.

8. Diez Roux AV, Mair C. Neighborhoods and health. Annals of the New York Academy of Sciences. 2010;1186(1):125-45. doi: 10.1111/j.1749-6632.2009.05333.x.

9. Brokamp C, Beck AF, Goyal NK, Ryan P, Greenberg JM, Hall ES. Material community deprivation and hospital utilization during the first year of life: an urban population-based cohort study. Ann Epidemiol. 2019;30:37-43. doi: 10.1016/j.annepidem.2018.11.008.

10. Wheeler DC, Raman S, Jones RM, Schootman M, Nelson EJ. Bayesian deprivation index models for explaining variation in elevated blood lead levels among children in Maryland. Spat Spatiotemporal Epidemiol. 2019;30:100286. doi: 10.1016/j.sste.2019.100286.

11. Yost K, Perkins C, Cohen R, Morris C, Wright W. Socioeconomic status and breast cancer incidence in California for different race/ethnic groups. Cancer Causes & Control. 2001;12(8):703-11. doi: 10.1023/A:1011240019516.

12. Ross CE, Mirowsky J. Neighborhood Disadvantage, Disorder, and Health. Journal of Health and Social Behavior. 2001;42(3):258-76. doi: 10.2307/3090214.

13. Family Budget Calculator. Economic Policy Institute.

14. O'Hare W, Mather M. The Growing Number of Kids in Severely Distressed Neighborhoods: Evidence from the 2000 Census. A Kids Count/PRB Report on Census 2000. Annie E; 2003.

15. Leventhal T, Brooks-Gunn J. The neighborhoods they live in: The effects of neighborhood residence on child and adolescent outcomes. Psychological Bulletin. 2000;126(2):309-37. doi: 10.1037/0033-2909.126.2.309.

16. Molnar BE, Miller MJ, Azrael D, Buka SL. Neighborhood Predictors of Concealed Firearm Carrying Among Children and Adolescents: Results From the Project on Human Development in Chicago Neighborhoods. Archives of Pediatrics & Adolescent Medicine. 2004;158(7):657-64. doi: 10.1001/archpedi.158.7.657.

17. Darden J, Rahbar M, Jezierski L, Li M, Velie E. The Measurement of Neighborhood Socioeconomic Characteristics and Black and White Residential Segregation in Metropolitan Detroit: Implications for the Study of Social Disparities in Health. Annals of the Association of American Geographers. 2010;100(1):137-58.

18. Bonito AJ, Bann C, Eicheldinger C, Carpenter L. Creation of New Race-Ethnicity Codes and Socioeconomic Status (SES) Indicators for Medicare Beneficiaries. Rockville, MD: Agency for Healthcare Research and Quality; 2008.

19. Lupo PJ, Danysh HE, Symanski E, Langlois PH, Cai Y, Swartz MD. Neighborhood-Based Socioeconomic Position and Risk of Oral Clefts Among Offspring. Am J Public Health. 2015;105(12):2518-25. doi: 10.2105/ajph.2015.302804.

20. Anderson S, Leventhal T, Dupéré V. Exposure to Neighborhood Affluence and Poverty in Childhood and Adolescence and Academic Achievement and Behavior. Applied Developmental Science. 2014;18(3):123-38. doi: 10.1080/10888691.2014.924355.

21. Coley SL, Nichols TR, Rulison KL, Aronson RE, Brown-Jeffy SL, Morrison SD. Does Neighborhood Risk Explain Racial Disparities in Low Birth Weight among Infants Born to Adolescent Mothers? J Pediatr Adolesc Gynecol. 2016;29(2):122-9. doi: 10.1016/j.jpag.2015.08.004.

22. California Healthy Places Index. Public Health Allliance of Southern California.

23. Cohen S, Prachand N, Bocksay K, Sayer J, Schuh T. Healthy Chicago 2.0 Community Health Assessment: Informing efforts to achieve health equity 2016-2020. Chicago: Chicago Department of Public Health; 2016.

24. Yang J, Schupp CW, Harrati A, Clarke C, Keegan THM, Gomez SL. Developing an area-based socioeconomic measure from American Community Survey Data. Freemont, CA: Cancer Prevention Institute of California; 2014.

25. Kesler P. Distressed Communities. Economic Innovation Group.

26. Montiel L, Nathan R, Wright D. An update on urban hardship. . Albany, NY: The Nelson A. Rockefeller Institute of Government; 2004.
