# Supplementary material for: Unpacking Neighborhood Socioeconomic Status in Children’s Health Research from an Environmental Justice Perspective: A Scoping Review
Source: Curr Environ Health Rep. 2024 Apr 10;11(2):288–99. doi: 10.1007/s40572-024-00445-8 (PMC11081999; doi:10.1007/s40572-024-00445-8)
Supplement: Supplementary file 2 — (DOCX 42 kb) [file 40572_2024_445_MOESM2_ESM.docx]

**Supplemental Appendix: Complete Reference List of Extracted Articles**

1. Adel Fahmideh M, Schraw JM, Chintagumpala M, Lupo PJ, Oluyomi AO, Scheurer ME. Neighborhood Socioeconomic Deprivation and Mortality in Children with Central Nervous System Tumors. Cancer Epidemiol Biomarkers Prev. 2021;30(12):2278-85. doi: 10.1158/1055-9965.Epi-21-0368.

2. Akbari SHA, Oates GR, Gonzalez-Sigler I, Arynchyna AA, McCroskey J, Alford EN, et al. Care for Chiari malformation type I: the role of socioeconomic disadvantage and race. J Neurosurg Pediatr. 2021:1-7. doi: 10.3171/2021.9.Peds21258.

3. Alishlash AS, Rutland SB, Friedman AJ, Hampton JI, Nourani A, Lebensburger J, et al. Acute chest syndrome in pediatric sickle cell disease: Associations with racial composition and neighborhood deprivation. Pediatr Blood Cancer. 2021;68(4):e28877. doi: 10.1002/pbc.28877.

4. Anderson S, Leventhal T, Dupéré V. Exposure to Neighborhood Affluence and Poverty in Childhood and Adolescence and Academic Achievement and Behavior. Applied Developmental Science. 2014;18(3):123-38. doi: 10.1080/10888691.2014.924355.

5. Angelidou A, Sullivan K, Melvin PR, Shui JE, Goldfarb IT, Bartolome R, et al. Association of Maternal Perinatal SARS-CoV-2 Infection With Neonatal Outcomes During the COVID-19 Pandemic in Massachusetts. JAMA Netw Open. 2021;4(4):e217523. doi: 10.1001/jamanetworkopen.2021.7523.

6. Aris IM, Rifas-Shiman SL, Jimenez MP, Li LJ, Hivert MF, Oken E, et al. Neighborhood Child Opportunity Index and Adolescent Cardiometabolic Risk. Pediatrics. 2021;147(2). doi: 10.1542/peds.2020-018903.

7. Austin MT, Hamilton E, Zebda D, Nguyen H, Eberth JM, Chang Y, et al. Health disparities and impact on outcomes in children with primary central nervous system solid tumors. J Neurosurg Pediatr. 2016;18(5):585-93. doi: 10.3171/2016.5.Peds15704.

8. Bagley EJ, Fuller-Rowell TE, Saini EK, Philbrook LE, El-Sheikh M. Neighborhood Economic Deprivation and Social Fragmentation: Associations With Children's Sleep. Behav Sleep Med. 2018;16(6):542-52. doi: 10.1080/15402002.2016.1253011.

9. Banwell E, Collaco JM, Oates GR, Rice JL, Juarez LD, Young LR, et al. Area deprivation and respiratory morbidities in children with bronchopulmonary dysplasia. Pediatr Pulmonol. 2022;57(9):2053-9. doi: 10.1002/ppul.25969.

10. Barboza-Salerno GE. Examining Spatial Regimes of Child Maltreatment Allegations in a Social Vulnerability Framework. Child Maltreat. 2020;25(1):70-84. doi: 10.1177/1077559519850340.

11. Barboza-Salerno GE. Variability and stability in child maltreatment risk across time and space and its association with neighborhood social & housing vulnerability in New Mexico: A bayesian space-time model. Child Abuse Negl. 2020;104:104472. doi: 10.1016/j.chiabu.2020.104472.

12. Barch DM, Albaugh MD, Baskin-Sommers A, Bryant BE, Clark DB, Dick AS, et al. Demographic and mental health assessments in the adolescent brain and cognitive development study: Updates and age-related trajectories. Dev Cogn Neurosci. 2021;52:101031. doi: 10.1016/j.dcn.2021.101031.

13. Barr PB. Early neighborhood conditions and trajectories of depressive symptoms across adolescence and into adulthood. Advances in Life Course Research. 2018;35:57-68. doi: https://doi.org/10.1016/j.alcr.2018.01.005.

14. Barr PB. Neighborhood conditions and trajectories of alcohol use and misuse across the early life course. Health & Place. 2018;51:36-44. doi: https://doi.org/10.1016/j.healthplace.2018.02.007.

15. Benmarhnia T, Huang J, Basu R, Wu J, Bruckner TA. Decomposition Analysis of Black-White Disparities in Birth Outcomes: The Relative Contribution of Air Pollution and Social Factors in California. Environ Health Perspect. 2017;125(10):107003. doi: 10.1289/ehp490.

16. Bishop-Royse J, Lange-Maia B, Murray L, Shah RC, DeMaio F. Structural racism, socio-economic marginalization, and infant mortality. Public Health. 2021;190:55-61. doi: 10.1016/j.puhe.2020.10.027.

17. Bleil ME, Appelhans BM, Thomas AS, Gregorich SE, Marquez N, Roisman GI, et al. Early life predictors of positive change during the coronavirus disease pandemic. BMC Psychol. 2021;9(1):83. doi: 10.1186/s40359-021-00586-7.

18. Bodnar C, Buss R, Somers K, Mokdad A, Van Arendonk KJ. Association of Neighborhood Socioeconomic Disadvantage With Complicated Appendicitis in Children. J Surg Res. 2021;265:245-51. doi: 10.1016/j.jss.2021.03.036.

19. Boynton SA, Matheson MB, Ng DK, Hidalgo G, Warady BA, Furth SL, et al. The Relationship Between Neighborhood Disadvantage and Kidney Disease Progression in the Chronic Kidney Disease in Children (CKiD) Cohort. Am J Kidney Dis. 2022;80(2):207-14. doi: 10.1053/j.ajkd.2021.12.008.

20. Brettin K, Shah AS, Welcher J, Jastrzembski B. Reduced visits to pediatric eye care among socioeconomically disadvantaged patients during the COVID-19 pandemic. J aapos. 2022;26(4):195-6. doi: 10.1016/j.jaapos.2022.03.006.

21. Brewer M, Kimbro RT. Neighborhood context and immigrant children's physical activity. Soc Sci Med. 2014;116:1-9. doi: 10.1016/j.socscimed.2014.06.022.

22. Brewer M, Kimbro RT, Denney JT, Osiecki KM, Moffett B, Lopez K. Does neighborhood social and environmental context impact race/ethnic disparities in childhood asthma? Health Place. 2017;44:86-93. doi: 10.1016/j.healthplace.2017.01.006.

23. Brick LAD, Nugent NR, Kahana SY, Bruce D, Tanney MR, Fernández MI, et al. Interaction Effects of Neighborhood Disadvantage and Individual Social Support on Frequency of Alcohol Use in Youth Living with HIV. Am J Community Psychol. 2018;61(3-4):276-84. doi: 10.1002/ajcp.12227.

24. Brody GH, Yu T, Miller GE, Ehrlich KB, Chen E. Preventive parenting intervention during childhood and young black adults' unhealthful behaviors: a randomized controlled trial. J Child Psychol Psychiatry. 2019;60(1):63-71. doi: 10.1111/jcpp.12968.

25. Brokamp C, Beck AF, Goyal NK, Ryan P, Greenberg JM, Hall ES. Material community deprivation and hospital utilization during the first year of life: an urban population-based cohort study. Ann Epidemiol. 2019;30:37-43. doi: 10.1016/j.annepidem.2018.11.008.

26. Brown LE, França UL, McManus ML. Socioeconomic Disadvantage and Distance to Pediatric Critical Care. Pediatr Crit Care Med. 2021;22(12):1033-41. doi: 10.1097/pcc.0000000000002807.

27. Bruckner TA, Kane JB, Gailey S. Strong upward neighborhood mobility and preterm birth: a matched-sibling design approach. Ann Epidemiol. 2019;36:48-54.e1. doi: 10.1016/j.annepidem.2019.05.005.

28. Bucholz EM, Sleeper LA, Newburger JW. Neighborhood Socioeconomic Status and Outcomes Following the Norwood Procedure: An Analysis of the Pediatric Heart Network Single Ventricle Reconstruction Trial Public Data Set. J Am Heart Assoc. 2018;7(3). doi: 10.1161/jaha.117.007065.

29. Byck GR, Bolland J, Dick D, Swann G, Henry D, Mustanski B. Effect of housing relocation and neighborhood environment on adolescent mental and behavioral health. J Child Psychol Psychiatry. 2015;56(11):1185-93. doi: 10.1111/jcpp.12386.

30. Carlson DL, McNulty TL, Bellair PE, Watts S. Neighborhoods and racial/ethnic disparities in adolescent sexual risk behavior. J Youth Adolesc. 2014;43(9):1536-49. doi: 10.1007/s10964-013-0052-0.

31. Carrillo G, Perez Patron MJ, Johnson N, Zhong Y, Lucio R, Xu X. Asthma prevalence and school-related hazardous air pollutants in the US-México border area. Environ Res. 2018;162:41-8. doi: 10.1016/j.envres.2017.11.057.

32. Carroll SL, Klump KL, Burt SA. Understanding the effects of neighborhood disadvantage on youth psychopathology. Psychol Med. 2022:1-11. doi: 10.1017/s0033291721005080.

33. Carroll-Scott A, Gilstad-Hayden K, Rosenthal L, Eldahan A, McCaslin C, Peters SM, et al. Associations of Neighborhood and School Socioeconomic and Social Contexts With Body Mass Index Among Urban Preadolescent Students. Am J Public Health. 2015;105(12):2496-502. doi: 10.2105/ajph.2015.302882.

34. Carroll-Scott A, Gilstad-Hayden K, Rosenthal L, Peters SM, McCaslin C, Joyce R, et al. Disentangling neighborhood contextual associations with child body mass index, diet, and physical activity: the role of built, socioeconomic, and social environments. Soc Sci Med. 2013;95:106-14. doi: 10.1016/j.socscimed.2013.04.003.

35. Castro IE, Hruska B, Gump BB. Race Differences in the Effect of Subjective Social Status on Hostility and Depressive Symptoms Among 9- to 11-Year-Old Children. J Racial Ethn Health Disparities. 2020;7(5):844-53. doi: 10.1007/s40615-020-00707-9.

36. Chang LY, Foshee VA, Reyes HL, Ennett ST, Halpern CT. Direct and indirect effects of neighborhood characteristics on the perpetration of dating violence across adolescence. J Youth Adolesc. 2015;44(3):727-44. doi: 10.1007/s10964-014-0190-z.

37. Cheung AY, Kan KY, Jang S, Cohen MB, Levi JR. Socioeconomic variables as a predictor of indication for pediatric adenotonsillectomy. Int J Pediatr Otorhinolaryngol. 2020;136:110181. doi: 10.1016/j.ijporl.2020.110181.

38. Chien NC, Mistry RS. Geographic variations in cost of living: associations with family and child well-being. Child Dev. 2013;84(1):209-25. doi: 10.1111/j.1467-8624.2012.01846.x.

39. Clennin MN, Pate RR. The Association Between Neighborhood Socioeconomic Deprivation, Cardiorespiratory Fitness, and Physical Activity in US Youth. Journal of Physical Activity and Health. 2019;16(12):1147-53. doi: 10.1123/jpah.2019-0039.

40. Coley SL, Nichols TR, Rulison KL, Aronson RE, Brown-Jeffy SL, Morrison SD. Does Neighborhood Risk Explain Racial Disparities in Low Birth Weight among Infants Born to Adolescent Mothers? J Pediatr Adolesc Gynecol. 2016;29(2):122-9. doi: 10.1016/j.jpag.2015.08.004.

41. Cook WK, Karriker-Jaffe KJ, Bond J, Lui C. Asian American problem drinking trajectories during the transition to adulthood: ethnic drinking cultures and neighborhood contexts. Am J Public Health. 2015;105(5):1020-7. doi: 10.2105/ajph.2014.302196.

42. Cooney T, Fisher PG, Tao L, Clarke CA, Partap S. Pediatric neuro-oncology survival disparities in California. J Neurooncol. 2018;138(1):83-97. doi: 10.1007/s11060-018-2773-0.

43. Crespi CM, Wang MC, Seto E, Mare R, Gee G. Associations of family and neighborhood socioeconomic characteristics with longitudinal adiposity patterns in a biracial cohort of adolescent girls. Biodemography Soc Biol. 2015;61(1):81-97. doi: 10.1080/19485565.2014.981794.

44. Curci SG, Hernández JC, Luecken LJ, Perez M. Multilevel prenatal socioeconomic determinants of Mexican American children's weight: Mediation by breastfeeding. Health Psychol. 2020;39(11):997-1006. doi: 10.1037/hea0001028.

45. D'Agostino EM, Patel HH, Hansen E, Mathew MS, Nardi MI, Messiah SE. Does transportation vulnerability explain the relationship between changes in exposure to segregation and youth cardiovascular health? Health Place. 2019;57:265-76. doi: 10.1016/j.healthplace.2019.04.002.

46. Daniels K, Lê-Scherban F, Auchincloss AH, Moore K, Melly S, Razzaghi H, et al. Longitudinal associations of neighborhood environment features with pediatric body mass index. Health Place. 2021;71:102656. doi: 10.1016/j.healthplace.2021.102656.

47. Daniels KM, Lê-Scherban F, Schinasi LH, Moore K, Auchincloss AH, Forrest CB, et al. Cross-Sectional Associations of Built and Social Neighborhood Environment Variables with Body Mass Index in a Large Sample of Urban Predominantly African American Children. Child Obes. 2021;17(3):209-19. doi: 10.1089/chi.2020.0155.

48. Davies RR, Russo MJ, Reinhartz O, Maeda K, Rosenthal DN, Chin C, et al. Lower socioeconomic status is associated with worse outcomes after both listing and transplanting children with heart failure. Pediatr Transplant. 2013;17(6):573-81. doi: 10.1111/petr.12117.

49. Dawson CT, Wu W, Fennie KP, Ibañez G, Cano M, Pettit JW, et al. Perceived neighborhood social cohesion moderates the relationship between neighborhood structural disadvantage and adolescent depressive symptoms. Health Place. 2019;56:88-98. doi: 10.1016/j.healthplace.2019.01.001.

50. Dawson CT, Wu W, Fennie KP, Ibañez G, Cano M, Pettit JW, et al. Parental-perceived neighborhood characteristics and adolescent depressive symptoms: A multilevel moderation analysis. J Community Psychol. 2019;47(7):1568-90. doi: 10.1002/jcop.22205.

51. de Cos V, Rooney AS, Sykes AG, Ghetti CB, Henry OS, Krzyzaniak A, et al. Integrating traffic safety data with area deprivation index: A method to better understand the causes of pediatric pedestrian versus automobile collisions. J Trauma Acute Care Surg. 2022;93(5):650-5. doi: 10.1097/ta.0000000000003666.

52. DeRouen MC, Parsons HM, Kent EE, Pollock BH, Keegan THM. Sociodemographic disparities in survival for adolescents and young adults with cancer differ by health insurance status. Cancer Causes Control. 2017;28(8):841-51. doi: 10.1007/s10552-017-0914-y.

53. Dionne A, Bucholz EM, Gauvreau K, Gould P, Son MBF, Baker AL, et al. Impact of Socioeconomic Status on Outcomes of Patients with Kawasaki Disease. J Pediatr. 2019;212:87-92. doi: 10.1016/j.jpeds.2019.05.024.

54. Discepolo K, Melvin P, Ghazarians M, Tennermann N, Ward VL. Socioeconomic and Clinical Demography of Dental Missed Care Opportunities. JDR Clin Trans Res. 2022:23800844221104790. doi: 10.1177/23800844221104790.

55. Ellis DA, Cutchin MP, Templin T, Carcone AI, Evans M, Weissberg-Benchell J, et al. Effects of family and neighborhood risks on glycemic control among young black adolescents with type 1 diabetes: Findings from a multi-center study. Pediatr Diabetes. 2021;22(3):511-8. doi: 10.1111/pedi.13176.

56. Estrada-Martínez LM, Cantrell C, Sewell W, Butler-Barnes S, Cabrera-Nguyen EP. Adolescent Neighborhood Environments and Latino Intraethnic Disparities in Trajectories of Serious Violent Behaviors. Hisp J Behav Sci. 2017;39(4):504-27. doi: 10.1177/0739986317734325.

57. Evans S, Okoroafor UC, Calfee RP. Is Social Deprivation Associated with PROMIS Outcomes After Upper Extremity Fractures in Children? Clin Orthop Relat Res. 2021;479(4):826-34. doi: 10.1097/corr.0000000000001571.

58. Fabricant PD, McLaren SH, Suryavanshi JR, Nwachukwu BU, Dodwell ER. Association Between Government Health Insurance Status and Physical Activity in American Youth. J Pediatr Orthop. 2019;39(7):e552-e7. doi: 10.1097/bpo.0000000000001329.

59. Fagan AA, Wright EM, Pinchevsky GM. The protective effects of neighborhood collective efficacy on adolescent substance use and violence following exposure to violence. J Youth Adolesc. 2014;43(9):1498-512. doi: 10.1007/s10964-013-0049-8.

60. Fagan AA, Wright EM, Pinchevsky GM. Exposure to violence, substance use, and neighborhood context. Soc Sci Res. 2015;49:314-26. doi: 10.1016/j.ssresearch.2014.08.015.

61. Fairman BJ, Goldstein RB, Simons-Morton BG, Haynie DL, Liu D, Hingson RW, et al. Neighbourhood context and binge drinking from adolescence into early adulthood in a US national cohort. Int J Epidemiol. 2020;49(1):103-12. doi: 10.1093/ije/dyz133.

62. Finch BK, Thomas K, Gibbons JR, Beck AN. The Impact of Residing in a Gang Territory on Adverse Birth Outcomes: Evidence from Los Angeles. J Urban Health. 2021;98(2):233-47. doi: 10.1007/s11524-020-00512-z.

63. Finegood ED, Rarick JRD, Blair C. Exploring longitudinal associations between neighborhood disadvantage and cortisol levels in early childhood. Dev Psychopathol. 2017;29(5):1649-62. doi: 10.1017/s0954579417001304.

64. Finlay E, Palmer S, Abes B, Abo B, Fishe JN. Clinical, Operational, and Socioeconomic Analysis of EMS Bypass of the Closest Facility for Pediatric Asthma Patients. West J Emerg Med. 2021;22(4):972-8. doi: 10.5811/westjem.2021.4.50382.

65. Flagg LA, Needham BL, Locher JL. Neighborhood Disadvantage, Preconception Health Behaviors and Infant Birthweight: A Preliminary Study. Int J Contemp Sociol. 2014;51(1):7-25.

66. Gard AM, Maxwell AM, Shaw DS, Mitchell C, Brooks-Gunn J, McLanahan SS, et al. Beyond family-level adversities: Exploring the developmental timing of neighborhood disadvantage effects on the brain. Dev Sci. 2021;24(1):e12985. doi: 10.1111/desc.12985.

67. George KM, Lutsey PL, Kucharska-Newton A, Palta P, Heiss G, Osypuk T, et al. Life-Course Individual and Neighborhood Socioeconomic Status and Risk of Dementia in the Atherosclerosis Risk in Communities Neurocognitive Study. Am J Epidemiol. 2020;189(10):1134-42. doi: 10.1093/aje/kwaa072.

68. Giddens NT, Juneau P, Manza P, Wiers CE, Volkow ND. Disparities in sleep duration among American children: effects of race and ethnicity, income, age, and sex. Proc Natl Acad Sci U S A. 2022;119(30):e2120009119. doi: 10.1073/pnas.2120009119.

69. Glazer KB, Eliot MN, Danilack VA, Carlson L, Phipps MG, Dadvand P, et al. Residential green space and birth outcomes in a coastal setting. Environ Res. 2018;163:97-107. doi: 10.1016/j.envres.2018.01.006.

70. Goldstein RB, Lee AK, Haynie DL, Luk JW, Fairman BJ, Liu D, et al. Neighbourhood disadvantage and depressive symptoms among adolescents followed into emerging adulthood. J Epidemiol Community Health. 2019;73(7):590-7. doi: 10.1136/jech-2018-212004.

71. Gonzales FA, Jones RR, Deardorff J, Windham GC, Hiatt RA, Kushi LH. Neighborhood deprivation, race/ethnicity, and urinary metal concentrations among young girls in California. Environ Int. 2016;91:29-39. doi: 10.1016/j.envint.2016.02.004.

72. Graham C, Reither EN, Ciciurkaite G, Dev DA, Fargo J. Does context matter? A multilevel analysis of neighborhood disadvantage and children's sleep health. Sleep Health. 2020;6(5):578-86. doi: 10.1016/j.sleh.2020.05.002.

73. Hackman DA, Cserbik D, Chen JC, Berhane K, Minaravesh B, McConnell R, et al. Association of Local Variation in Neighborhood Disadvantage in Metropolitan Areas With Youth Neurocognition and Brain Structure. JAMA Pediatr. 2021;175(8):e210426. doi: 10.1001/jamapediatrics.2021.0426.

74. Hails KA, Shaw DS. Associations Between Boys' Early Childhood Exposure to Family and Neighborhood Poverty and Body Mass Index in Early Adolescence. J Pediatr Psychol. 2019;44(9):1009-18. doi: 10.1093/jpepsy/jsz047.

75. Heffernan MJ, Younis M, Song B, Fontenot B, Dewitz R, Brooks JT, et al. Disparities in Pediatric Scoliosis: The Impact of Race and Insurance Type on Access to Nonoperative Treatment for Adolescent Idiopathic Scoliosis. J Pediatr Orthop. 2022;42(8):427-31. doi: 10.1097/bpo.0000000000002213.

76. Hegde ST, Wagner AL, Clarke PJ, Potter RC, Swanson RG, Boulton ML. Neighbourhood influence on the fourth dose of diphtheria-tetanus-pertussis vaccination. Public Health. 2019;167:41-9. doi: 10.1016/j.puhe.2018.11.009.

77. Henry OS, Rooney AS, Heflinger MV, Sykes AG, Ghetti CB, de Cos V, et al. Bike Helmet Usage in the Most Disadvantaged Neighborhoods: A Focused Area for Trauma Prevention. J Surg Res. 2022;278:7-13. doi: 10.1016/j.jss.2022.04.033.

78. Hoke MK, Boen CE. The health impacts of eviction: Evidence from the national longitudinal study of adolescent to adult health. Soc Sci Med. 2021;273:113742. doi: 10.1016/j.socscimed.2021.113742.

79. Huang JH, Hipp JA, Marquet O, Alberico C, Fry D, Mazak E, et al. Neighborhood characteristics associated with park use and park-based physical activity among children in low-income diverse neighborhoods in New York City. Prev Med. 2020;131:105948. doi: 10.1016/j.ypmed.2019.105948.

80. Huang Y, Edwards J, Laurel-Wilson M. The shadow of context: Neighborhood and school socioeconomic disadvantage, perceived social integration, and the mental and behavioral health of adolescents. Health Place. 2020;66:102425. doi: 10.1016/j.healthplace.2020.102425.

81. Huynh M, Maroko AR. Gentrification and preterm birth in New York City, 2008–2010. J Urban Health. 2014;91(1):211-20. doi: 10.1007/s11524-013-9823-x.

82. Jennings JM, Hensel DJ, Tanner AE, Reilly ML, Ellen JM. Are social organizational factors independently associated with a current bacterial sexually transmitted infection among urban adolescents and young adults? Soc Sci Med. 2014;118:52-60. doi: 10.1016/j.socscimed.2014.07.062.

83. Jimenez MP, Wellenius GA, Subramanian SV, Buka S, Eaton C, Gilman SE, et al. Longitudinal associations of neighborhood socioeconomic status with cardiovascular risk factors: A 46-year follow-up study. Soc Sci Med. 2019;241:112574. doi: 10.1016/j.socscimed.2019.112574.

84. Johnson KA, Snyder RE, Tang EC, de Guzman NS, Plotzker RE, Murphy R, et al. Geospatial Social Determinants of Health Correlate with Disparities in Syphilis and Congenital Syphilis Cases in California. Pathogens. 2022;11(5). doi: 10.3390/pathogens11050547.

85. Kahana SY, Jenkins RA, Bruce D, Fernandez MI, Hightow-Weidman LB, Bauermeister JA. Structural Determinants of Antiretroviral Therapy Use, HIV Care Attendance, and Viral Suppression among Adolescents and Young Adults Living with HIV. PLoS One. 2016;11(4):e0151106. doi: 10.1371/journal.pone.0151106.

86. Kent EE, Sender LS, Morris RA, Grigsby TJ, Montoya MJ, Ziogas A, et al. Multilevel socioeconomic effects on quality of life in adolescent and young adult survivors of leukemia and lymphoma. Qual Life Res. 2013;22(6):1339-51. doi: 10.1007/s11136-012-0254-z.

87. Kersten EE, Adler NE, Gottlieb L, Jutte DP, Robinson S, Roundfield K, et al. Neighborhood Child Opportunity and Individual-Level Pediatric Acute Care Use and Diagnoses. Pediatrics. 2018;141(5). doi: 10.1542/peds.2017-2309.

88. Kim Y, Landgraf A, Colabianchi N. Living in High-SES Neighborhoods Is Protective against Obesity among Higher-Income Children but Not Low-Income Children: Results from the Healthy Communities Study. J Urban Health. 2020;97(2):175-90. doi: 10.1007/s11524-020-00427-9.

89. Kowaleski-Jones L, Fan JX, Wen M, Hanson H. Neighborhood Context and Youth Physical Activity: Differential Associations by Gender and Age. Am J Health Promot. 2017;31(5):426-34. doi: 10.1177/0890117116667353.

90. Kravitz-Wirtz N. Cumulative Effects of Growing Up in Separate and Unequal Neighborhoods on Racial Disparities in Self-rated Health in Early Adulthood. J Health Soc Behav. 2016;57(4):453-70. doi: 10.1177/0022146516671568.

91. Kravitz-Wirtz N. Temporal Effects of Child and Adolescent Exposure to Neighborhood Disadvantage on Black/White Disparities in Young Adult Obesity. J Adolesc Health. 2016;58(5):551-7. doi: 10.1016/j.jadohealth.2016.01.004.

92. Kucik JE, Nembhard WN, Donohue P, Devine O, Wang Y, Minkovitz CS, et al. Community socioeconomic disadvantage and the survival of infants with congenital heart defects. Am J Public Health. 2014;104(11):e150-7. doi: 10.2105/ajph.2014.302099.

93. Kurani S, MacLaughlin KL, Jacobson RM, St Sauver JL, Jenkins GD, Fan C, et al. Socioeconomic disadvantage and human papillomavirus (HPV) vaccination uptake. Vaccine. 2022;40(3):471-6. doi: 10.1016/j.vaccine.2021.12.003.

94. Lara-Cinisomo S, Xue Y, Brooks-Gunn J. Latino youth's internalising behaviours: links to immigrant status and neighbourhood characteristics. Ethn Health. 2013;18(3):315-35. doi: 10.1080/13557858.2012.734278.

95. Lean RE, Gerstein ED, Smyser TA, Smyser CD, Rogers CE. Socioeconomic disadvantage and parental mood/affective problems links negative parenting and executive dysfunction in children born very preterm. Dev Psychopathol. 2021:1-16. doi: 10.1017/s0954579421000961.

96. Lee H, Estrada-Martínez LM. Trajectories of Depressive Symptoms and Neighborhood Changes from Adolescence to Adulthood: Latent Class Growth Analysis and Multilevel Growth Curve Models. Int J Environ Res Public Health. 2020;17(6). doi: 10.3390/ijerph17061829.

97. Lei MK, Simons RL, Simons LG, Edmond MB. Gender equality and violent behavior: how neighborhood gender equality influences the gender gap in violence. Violence Vict. 2014;29(1):89-108. doi: 10.1891/0886-6708.vv-d-12-00102.

98. Liao K, Chorney SR, Brown AB, Brooks RL, Sewell A, Bailey C, et al. The Impact of Socioeconomic Disadvantage on Pediatric Tracheostomy Outcomes. Laryngoscope. 2021;131(11):2603-9. doi: 10.1002/lary.29576.

99. Link-Gelles R, Westreich D, Aiello AE, Shang N, Weber DJ, Rosen JB, et al. Generalisability of vaccine effectiveness estimates: an analysis of cases included in a postlicensure evaluation of 13-valent pneumococcal conjugate vaccine in the USA. BMJ Open. 2017;7(8):e017715. doi: 10.1136/bmjopen-2017-017715.

100. Lopez-Tamayo R, LaVome Robinson W, Lambert SF, Jason LA, Ialongo NS. Parental Monitoring, Association with Externalized Behavior, and Academic Outcomes in Urban African-American Youth: A Moderated Mediation Analysis. Am J Community Psychol. 2016;57(3-4):366-79. doi: 10.1002/ajcp.12056.

101. Lothrop N, Hussaini K, Billheimer D, Beamer P. Community-level characteristics and environmental factors of child respiratory illnesses in Southern Arizona. BMC Public Health. 2017;17(1):516. doi: 10.1186/s12889-017-4424-3.

102. Lowe AA, Gerald JK, Clemens C, Gerald LB. School-based Stock Inhaler Programs and Neighborhood Disadvantage. J Health Care Poor Underserved. 2022;33(2):1083-93. doi: 10.1353/hpu.2022.0082.

103. Lupini F, Leichman ES, Gould RA, Walters RM, Mindell JA, Williamson AA. Correlates of a caregiver-reported child sleep problem and variation by community disadvantage. Sleep Med. 2022;90:83-90. doi: 10.1016/j.sleep.2022.01.009.

104. Lupo PJ, Danysh HE, Symanski E, Langlois PH, Cai Y, Swartz MD. Neighborhood-Based Socioeconomic Position and Risk of Oral Clefts Among Offspring. Am J Public Health. 2015;105(12):2518-25. doi: 10.2105/ajph.2015.302804.

105. Ma X, Fleischer NL, Liu J, Hardin JW, Zhao G, Liese AD. Neighborhood deprivation and preterm birth: an application of propensity score matching. Ann Epidemiol. 2015;25(2):120-5. doi: 10.1016/j.annepidem.2014.11.021.

106. Madkour AS, Harville EW, Xie Y. Neighborhood disadvantage, racial concentration and the birthweight of infants born to adolescent mothers. Matern Child Health J. 2014;18(3):663-71. doi: 10.1007/s10995-013-1291-0.

107. Manduca R, Sampson RJ. Childhood exposure to polluted neighborhood environments and intergenerational income mobility, teenage birth, and incarceration in the USA. Population and Environment. 2021;42(4):501-23. doi: 10.1007/s11111-020-00371-5.

108. Manickam S, Vivier PM, Rogers ML, McGowan EC, Smego R, Tucker R, et al. Neighborhood Inequality and Emergency Department Use in Neonatal Intensive Care Unit Graduates. J Pediatr. 2020;226:294-8.e1. doi: 10.1016/j.jpeds.2020.06.074.

109. Marshall NA, Marusak HA, Sala-Hamrick KJ, Crespo LM, Rabinak CA, Thomason ME. Socioeconomic disadvantage and altered corticostriatal circuitry in urban youth. Hum Brain Mapp. 2018;39(5):1982-94. doi: 10.1002/hbm.23978.

110. Martenies SE, Zhang M, Corrigan AE, Kvit A, Shields T, Wheaton W, et al. Associations between combined exposure to environmental hazards and social stressors at the neighborhood level and individual perinatal outcomes in the ECHO-wide cohort. Health Place. 2022;76:102858. doi: 10.1016/j.healthplace.2022.102858.

111. Martin CL, Kane JB, Miles GL, Aiello AE, Harris KM. Neighborhood disadvantage across the transition from adolescence to adulthood and risk of metabolic syndrome. Health Place. 2019;57:131-8. doi: 10.1016/j.healthplace.2019.03.002.

112. Martinez W, Polo AJ. Neighborhood Context, Family Cultural Values, and Latinx Youth Externalizing Problems. J Youth Adolesc. 2018;47(11):2440-52. doi: 10.1007/s10964-018-0914-6.

113. Mason MJ, Light JM, Mennis J, Rusby JC, Westling E, Crewe S, et al. Neighborhood disorder, peer network health, and substance use among young urban adolescents. Drug Alcohol Depend. 2017;178:208-14. doi: 10.1016/j.drugalcdep.2017.05.005.

114. McDonald TC, Heffernan MJ, Ramo B, Haber L, Sheffer B, Murphy J, et al. Surgical Outcomes of Obese Patients With Adolescent Idiopathic Scoliosis From Endemic Areas of Obesity in the United States. J Pediatr Orthop. 2021;41(10):e865-e70. doi: 10.1097/bpo.0000000000001958.

115. McManus BM, Robert S, Albanese A, Sadek-Badawi M, Palta M. Predictors of receiving therapy among very low birth weight 2-year olds eligible for Part C early intervention in Wisconsin. BMC Pediatr. 2013;13:106. doi: 10.1186/1471-2431-13-106.

116. Mehra R, Shebl FM, Cunningham SD, Magriples U, Barrette E, Herrera C, et al. Area-level deprivation and preterm birth: results from a national, commercially-insured population. BMC Public Health. 2019;19(1):236. doi: 10.1186/s12889-019-6533-7.

117. Melix BL, Uejio CK, Kintziger KW, Reid K, Duclos C, Jordan MM, et al. Florida neighborhood analysis of social determinants and their relationship to life expectancy. BMC Public Health. 2020;20(1):632. doi: 10.1186/s12889-020-08754-x.

118. Mennis J, McKeon TP, Coatsworth JD, Russell MA, Coffman DL, Mason MJ. Neighborhood disadvantage moderates the effect of a mobile health intervention on adolescent depression. Health Place. 2022;73:102728. doi: 10.1016/j.healthplace.2021.102728.

119. Meredith WJ, Cardenas-Iniguez C, Berman MG, Rosenberg MD. Effects of the physical and social environment on youth cognitive performance. Dev Psychobiol. 2022;64(4):e22258. doi: 10.1002/dev.22258.

120. Meza BC, Iacone D, Talwar D, Sankar WN, Shah AS. Socioeconomic Deprivation and Its Adverse Association with Adolescent Fracture Care Compliance. JB JS Open Access. 2020;5(2):e0064. doi: 10.2106/jbjs.Oa.19.00064.

121. Molina AL, Molina Y, Walley SC, Wu CL, Zhu A, Oates GR. Residential instability, neighborhood deprivation, and pediatric asthma outcomes. Pediatr Pulmonol. 2020;55(6):1340-8. doi: 10.1002/ppul.24771.

122. Moody HA, Darden JT, Pigozzi BW. The Relationship of Neighborhood Socioeconomic Differences and Racial Residential Segregation to Childhood Blood Lead Levels in Metropolitan Detroit. J Urban Health. 2016;93(5):820-39. doi: 10.1007/s11524-016-0071-8.

123. Morris MC, Bruehl S, Stone AL, Garber J, Smith C, Palermo TM, et al. Place and Pain: Association Between Neighborhood SES and Quantitative Sensory Testing Responses in Youth With Functional Abdominal Pain. J Pediatr Psychol. 2022;47(4):446-55. doi: 10.1093/jpepsy/jsab113.

124. Mudd AE, Michael YL, Melly S, Moore K, Diez-Roux A, Forrest CB. Spatial accessibility to pediatric primary care in Philadelphia: an area-level cross sectional analysis. Int J Equity Health. 2019;18(1):76. doi: 10.1186/s12939-019-0962-x.

125. Musa GJ, Geronazzo-Alman L, Fan B, Cheslack-Postava K, Bavley R, Wicks J, et al. Neighborhood characteristics and psychiatric disorders in the aftermath of mass trauma: A representative study of New York City public school 4th-12th graders after 9/11. J Psychiatr Res. 2021;138:584-90. doi: 10.1016/j.jpsychires.2021.05.002.

126. Nacht CL, Kelly MM, Edmonson MB, Sklansky DJ, Shadman KA, Kind AJH, et al. Association Between Neighborhood Disadvantage and Pediatric Readmissions. Matern Child Health J. 2022;26(1):31-41. doi: 10.1007/s10995-021-03310-4.

127. Nahmod NG, Master L, McClintock HF, Hale L, Buxton OM. Neighborhood Disadvantage Is Associated with Lower Quality Sleep and More Variability in Sleep Duration among Urban Adolescents. J Urban Health. 2022;99(1):102-15. doi: 10.1007/s11524-021-00570-x.

128. Nardone AL, Casey JA, Rudolph KE, Karasek D, Mujahid M, Morello-Frosch R. Associations between historical redlining and birth outcomes from 2006 through 2015 in California. PLoS One. 2020;15(8):e0237241. doi: 10.1371/journal.pone.0237241.

129. Nau C, Schwartz BS, Bandeen-Roche K, Liu A, Pollak J, Hirsch A, et al. Community socioeconomic deprivation and obesity trajectories in children using electronic health records. Obesity (Silver Spring). 2015;23(1):207-12. doi: 10.1002/oby.20903.

130. Nezwek TA, Braun SV, Menendez ME, Grussing ED, Shabin ZM. Area Deprivation Index and Magnitude of Scoliosis at Presentation to a Tertiary Referral Scoliosis Clinic in Massachusetts. J Pediatr Orthop. 2021;41(9):e712-e6. doi: 10.1097/bpo.0000000000001869.

131. Nichols OI, Fuller-Rowell TE, Robinson AT, Eugene D, Homandberg LK. Neighborhood Socioeconomic Deprivation in Early Childhood Mediates Racial Disparities in Blood Pressure in a College Student Sample. J Youth Adolesc. 2022;51(11):2146-60. doi: 10.1007/s10964-022-01658-6.

132. Nikulina V, Widom CS. Do race, neglect, and childhood poverty predict physical health in adulthood? A multilevel prospective analysis. Child Abuse Negl. 2014;38(3):414-24. doi: 10.1016/j.chiabu.2013.09.007.

133. Niu L, Hoyt LT, Pickering S, Nucci-Sack A, Salandy A, Shankar V, et al. Neighborhood Profiles and Body Mass Index Trajectory in Female Adolescents and Young Adults. J Adolesc Health. 2021;69(6):1024-31. doi: 10.1016/j.jadohealth.2021.06.010.

134. O'Lenick CR, Chang HH, Kramer MR, Winquist A, Mulholland JA, Friberg MD, et al. Ozone and childhood respiratory disease in three US cities: evaluation of effect measure modification by neighborhood socioeconomic status using a Bayesian hierarchical approach. Environ Health. 2017;16(1):36. doi: 10.1186/s12940-017-0244-2.

135. O'Lenick CR, Winquist A, Chang HH, Kramer MR, Mulholland JA, Grundstein A, et al. Evaluation of individual and area-level factors as modifiers of the association between warm-season temperature and pediatric asthma morbidity in Atlanta, GA. Environ Res. 2017;156:132-44. doi: 10.1016/j.envres.2017.03.021.

136. O'Lenick CR, Winquist A, Mulholland JA, Friberg MD, Chang HH, Kramer MR, et al. Assessment of neighbourhood-level socioeconomic status as a modifier of air pollution-asthma associations among children in Atlanta. J Epidemiol Community Health. 2017;71(2):129-36. doi: 10.1136/jech-2015-206530.

137. Oates GR, Harris WT, Rowe SM, Solomon GM, Dey S, Zhu A, et al. Area Deprivation as a Risk Factor for Methicillin-resistant Staphylococcus aureus Infection in Pediatric Cystic Fibrosis. Pediatr Infect Dis J. 2019;38(11):e285-e9. doi: 10.1097/inf.0000000000002419.

138. Oates GR, Rutland S, Juarez L, Friedman A, Schechter MS. The association of area deprivation and state child health with respiratory outcomes of pediatric patients with cystic fibrosis in the United States. Pediatr Pulmonol. 2021;56(5):883-90. doi: 10.1002/ppul.25192.

139. Okoroafor UC, Gerull W, Wright M, Guattery J, Sandvall B, Calfee RP. The Impact of Social Deprivation on Pediatric PROMIS Health Scores After Upper Extremity Fracture. J Hand Surg Am. 2018;43(10):897-902. doi: 10.1016/j.jhsa.2018.06.119.

140. Osypuk TL, Kehm R, Misra DP. Where we used to live: validating retrospective measures of childhood neighborhood context for life course epidemiologic studies. PLoS One. 2015;10(4):e0124635. doi: 10.1371/journal.pone.0124635.

141. Pabayo R, Cook DM, Farmer G, Molnar BE. Neighborhood Income Inequality and Alcohol Use among Adolescents in Boston, Massachusetts. Int J Environ Res Public Health. 2021;18(16). doi: 10.3390/ijerph18168484.

142. Pabayo R, Molnar BE, Cradock A, Kawachi I. The relationship between neighborhood socioeconomic characteristics and physical inactivity among adolescents living in Boston, Massachusetts. Am J Public Health. 2014;104(11):e142-9. doi: 10.2105/ajph.2014.302109.

143. Pabayo R, Molnar BE, Kawachi I. The role of neighborhood income inequality in adolescent aggression and violence. J Adolesc Health. 2014;55(4):571-9. doi: 10.1016/j.jadohealth.2014.04.012.

144. Pabayo R, Molnar BE, Street N, Kawachi I. The relationship between social fragmentation and sleep among adolescents living in Boston, Massachusetts. J Public Health (Oxf). 2014;36(4):587-98. doi: 10.1093/pubmed/fdu001.

145. Padula AM, Yang W, Carmichael SL, Tager IB, Lurmann F, Hammond SK, et al. Air Pollution, Neighbourhood Socioeconomic Factors, and Neural Tube Defects in the San Joaquin Valley of California. Paediatr Perinat Epidemiol. 2015;29(6):536-45. doi: 10.1111/ppe.12244.

146. Paksarian D, Rudolph KE, Stapp EK, Dunster GP, He J, Mennitt D, et al. Association of Outdoor Artificial Light at Night With Mental Disorders and Sleep Patterns Among US Adolescents. JAMA Psychiatry. 2020;77(12):1266-75. doi: 10.1001/jamapsychiatry.2020.1935.

147. Patterson JA, Keuler NS, Buckingham WR. Differences in Exclusive Breastfeeding Rates in US Hospitals According to Baby-Friendly Hospital Initiative Designation and Area Deprivation Index Category. Breastfeed Med. 2021;16(10):799-806. doi: 10.1089/bfm.2021.0050.

148. Pavela G. Is Childhood Socioeconomic Status Independently Associated with Adult BMI after Accounting for Adult and Neighborhood Socioeconomic Status? PLoS One. 2017;12(1):e0168481. doi: 10.1371/journal.pone.0168481.

149. Pharr J, Lough NL. Considering sport participation as a source for physical activity among adolescents. J Phys Act Health. 2014;11(5):930-41. doi: 10.1123/jpah.2012-0238.

150. Phillips GS, Wise LA, Rich-Edwards JW, Stampfer MJ, Rosenberg L. Neighborhood socioeconomic status in relation to preterm birth in a U.S. cohort of black women. J Urban Health. 2013;90(2):197-211. doi: 10.1007/s11524-012-9739-x.

151. Pourebrahim N, Shah P, VoPham T, Doody DR, Bell TR, deHart MP, et al. Time and geographic variations in human papillomavirus vaccine uptake in Washington state. Prev Med. 2021;153:106753. doi: 10.1016/j.ypmed.2021.106753.

152. Powers RJ, Mokdad AA, Pezzin LE, Nattinger AB, Oldham KT, Van Arendonk KJ. Disparities in utilization of outpatient surgical care among children. Surgery. 2021;170(6):1815-21. doi: 10.1016/j.surg.2021.05.045.

153. Puthenpura V, Du N, Hauptman L, Porto AF. Disparities in Telehealth Utilization Within Pediatric Gastroenterology: One Academic Center's Experience. J Pediatr Gastroenterol Nutr. 2021;73(2):217-22. doi: 10.1097/mpg.0000000000003167.

154. Putnam-Hornstein E, Prindle JJ, Rebbe R. Community disadvantage, family socioeconomic status, and racial/ethnic differences in maltreatment reporting risk during infancy. Child Abuse Negl. 2022;130(Pt 4):105446. doi: 10.1016/j.chiabu.2021.105446.

155. Queen TL, Baucom KJW, Baker AC, Mello D, Berg CA, Wiebe DJ. Neighborhood disorder and glycemic control in late adolescents with Type 1 diabetes. Soc Sci Med. 2017;183:126-9. doi: 10.1016/j.socscimed.2017.04.052.

156. Rakesh D, Zalesky A, Whittle S. Similar but distinct - Effects of different socioeconomic indicators on resting state functional connectivity: Findings from the Adolescent Brain Cognitive Development (ABCD) Study®. Dev Cogn Neurosci. 2021;51:101005. doi: 10.1016/j.dcn.2021.101005.

157. Rambo A, Rhodes L, Lomax J, Cao X, Steele J, Romer K, et al. Factors that Influence Acquisition of Lower Extremity Braces in the Pediatric Orthopaedic Population. J Pediatr Orthop. 2022;42(1):40-6. doi: 10.1097/bpo.0000000000001998.

158. Ramgopal S, Jaeger L, Cercone A, Martin-Gill C, Fishe J. The Child Opportunity Index and Pediatric Emergency Medical Services Utilization. Prehosp Emerg Care. 2023;27(2):238-45. doi: 10.1080/10903127.2022.2076268.

159. Ramphal B, DeSerisy M, Pagliaccio D, Raffanello E, Rauh V, Tau G, et al. Associations between Amygdala-Prefrontal Functional Connectivity and Age Depend on Neighborhood Socioeconomic Status. Cereb Cortex Commun. 2020;1(1):tgaa033. doi: 10.1093/texcom/tgaa033.

160. Ramphal B, Whalen DJ, Kenley JK, Yu Q, Smyser CD, Rogers CE, et al. Brain connectivity and socioeconomic status at birth and externalizing symptoms at age 2 years. Dev Cogn Neurosci. 2020;45:100811. doi: 10.1016/j.dcn.2020.100811.

161. Ray KN, Yahner KA, Bey J, Martin KC, Planey AM, Culyba AJ, et al. Understanding Variation In Nonurgent Pediatric Emergency Department Use In Communities With Concentrated Disadvantage. Health Aff (Millwood). 2021;40(1):156-64. doi: 10.1377/hlthaff.2020.00675.

162. Riley B, Schoeny M, Rogers L, Asiodu IV, Bigger HR, Meier PP, et al. Barriers to Human Milk Feeding at Discharge of Very Low-Birthweight Infants: Evaluation of Neighborhood Structural Factors. Breastfeed Med. 2016;11(7):335-42. doi: 10.1089/bfm.2015.0185.

163. Robinson MR, Daniel LC, O'Hara EA, Szabo MM, Barakat LP. Insurance status as a sociodemographic risk factor for functional outcomes and health-related quality of life among youth with sickle cell disease. J Pediatr Hematol Oncol. 2014;36(1):51-6. doi: 10.1097/mph.0000000000000013.

164. Rossen LM. Neighbourhood economic deprivation explains racial/ethnic disparities in overweight and obesity among children and adolescents in the U.S.A. J Epidemiol Community Health. 2014;68(2):123-9. doi: 10.1136/jech-2012-202245.

165. Rossen LM, Talih M. Social determinants of disparities in weight among US children and adolescents. Ann Epidemiol. 2014;24(10):705-13.e2. doi: 10.1016/j.annepidem.2014.07.010.

166. Roubinov DS, Hagan MJ, Boyce WT, Adler NE, Bush NR. Family Socioeconomic Status, Cortisol, and Physical Health in Early Childhood: The Role of Advantageous Neighborhood Characteristics. Psychosom Med. 2018;80(5):492-501. doi: 10.1097/psy.0000000000000585.

167. Rudolph KE, Stuart EA, Glass TA, Merikangas KR. Neighborhood disadvantage in context: the influence of urbanicity on the association between neighborhood disadvantage and adolescent emotional disorders. Soc Psychiatry Psychiatr Epidemiol. 2014;49(3):467-75. doi: 10.1007/s00127-013-0725-8.

168. Santiago AM, Berg KA, Leroux J. Assessing the Impact of Neighborhood Conditions on Neurodevelopmental Disorders during Childhood. Int J Environ Res Public Health. 2021;18(17). doi: 10.3390/ijerph18179041.

169. Schmidt NM, Glymour MM, Osypuk TL. Does the Temporal Pattern of Moving to a Higher-Quality Neighborhood Across a 5-Year Period Predict Psychological Distress Among Adolescents? Results From a Federal Housing Experiment. Am J Epidemiol. 2021;190(6):998-1008. doi: 10.1093/aje/kwaa256.

170. Schroeder K, Dumenci L, Sarwer DB, Noll JG, Henry KA, Suglia SF, et al. The Intersection of Neighborhood Environment and Adverse Childhood Experiences: Methods for Creation of a Neighborhood ACEs Index. Int J Environ Res Public Health. 2022;19(13). doi: 10.3390/ijerph19137819.

171. Schuck AM, Widom CS. Posttraumatic Stress Disorder in Maltreated Children Grown Up: The Influence of Neighborhood. J Trauma Stress. 2019;32(1):78-87. doi: 10.1002/jts.22355.

172. Sharma P, Kamath C, Kurani S, Pazdernik V, Kremers HM, St Sauver J, et al. Longitudinal Correlates of Increased Alcohol Use Among Adolescents and Young Adults During the COVID-19 Pandemic. Alcohol Alcohol. 2022;57(6):648-55. doi: 10.1093/alcalc/agac024.

173. Sheehan DM, Fennie KP, Mauck DE, Maddox LM, Lieb S, Trepka MJ. Retention in HIV Care and Viral Suppression: Individual- and Neighborhood-Level Predictors of Racial/Ethnic Differences, Florida, 2015. AIDS Patient Care STDS. 2017;31(4):167-75. doi: 10.1089/apc.2016.0197.

174. Slopen N, Non A, Williams DR, Roberts AL, Albert MA. Childhood adversity, adult neighborhood context, and cumulative biological risk for chronic diseases in adulthood. Psychosom Med. 2014;76(7):481-9. doi: 10.1097/psy.0000000000000081.

175. Song N, Sim JA, Dong Q, Zheng Y, Hou L, Li Z, et al. Blood DNA methylation signatures are associated with social determinants of health among survivors of childhood cancer. Epigenetics. 2022;17(11):1389-403. doi: 10.1080/15592294.2022.2030883.

176. Swisher RR, Warner TD. If They Grow up: Exploring the Neighborhood Context of Adolescent and Young Adult Survival Expectations. J Res Adolesc. 2013;23(4). doi: 10.1111/jora.12027.

177. Sykes AG, Rooney AS, Avila AG, Ghetti CB, Martin MJ, Bansal V, et al. Pediatric trauma in the California-Mexico border region: Injury disparities by Area Deprivation Index. J Trauma Acute Care Surg. 2022;92(5):831-8. doi: 10.1097/ta.0000000000003553.

178. Taylor RL, Cooper SR, Jackson JJ, Barch DM. Assessment of Neighborhood Poverty, Cognitive Function, and Prefrontal and Hippocampal Volumes in Children. JAMA Netw Open. 2020;3(11):e2023774. doi: 10.1001/jamanetworkopen.2020.23774.

179. Thomson J, Butts B, Camara S, Rasnick E, Brokamp C, Heyd C, et al. Neighborhood Socioeconomic Deprivation and Health Care Utilization of Medically Complex Children. Pediatrics. 2022;149(4). doi: 10.1542/peds.2021-052592.

180. Tomayko EJ, Weinert BA, Godfrey L, Adams AK, Hanrahan LP. Using Electronic Health Records to Examine Disease Risk in Small Populations: Obesity Among American Indian Children, Wisconsin, 2007-2012. Prev Chronic Dis. 2016;13:E29. doi: 10.5888/pcd13.150479.

181. Trinidad S, Brokamp C, Sahay R, Moody S, Gardner D, Parsons AA, et al. Children from disadvantaged neighborhoods experience disproportionate injury from interpersonal violence. J Pediatr Surg. 2023;58(3):545-51. doi: 10.1016/j.jpedsurg.2022.05.023.

182. Triplett RL, Lean RE, Parikh A, Miller JP, Alexopoulos D, Kaplan S, et al. Association of Prenatal Exposure to Early-Life Adversity With Neonatal Brain Volumes at Birth. JAMA Netw Open. 2022;5(4):e227045. doi: 10.1001/jamanetworkopen.2022.7045.

183. Vargas T, Damme KSF, Mittal VA. Neighborhood deprivation, prefrontal morphology and neurocognition in late childhood to early adolescence. Neuroimage. 2020;220:117086. doi: 10.1016/j.neuroimage.2020.117086.

184. Veras Y, Rogers ML, Smego R, Zonfrillo MR, Mello MJ, Vivier PM. Neighborhood Risk Factors for Pediatric Fall-Related Injuries: A Retrospective Analysis of a Statewide Hospital Network. Acad Pediatr. 2019;19(6):677-83. doi: 10.1016/j.acap.2018.11.012.

185. Vesoulis ZA, Lust CE, Cohlan BA, Liao SM, Mathur AM. Poverty and Excess Length of Hospital Stay in Neonatal Opioid Withdrawal Syndrome. J Addict Med. 2020;14(2):113-8. doi: 10.1097/adm.0000000000000540.

186. Vogel M, Van Ham M. Unpacking the Relationships between Impulsivity, Neighborhood Disadvantage, and Adolescent Violence: An Application of a Neighborhood-Based Group Decomposition. J Youth Adolesc. 2018;47(4):859-71. doi: 10.1007/s10964-017-0695-3.

187. Wadhwani SI, Beck AF, Bucuvalas J, Gottlieb L, Kotagal U, Lai JC. Neighborhood socioeconomic deprivation is associated with worse patient and graft survival following pediatric liver transplantation. Am J Transplant. 2020;20(6):1597-605. doi: 10.1111/ajt.15786.

188. Wadhwani SI, Bucuvalas JC, Brokamp C, Anand R, Gupta A, Taylor S, et al. Association Between Neighborhood-level Socioeconomic Deprivation and the Medication Level Variability Index for Children Following Liver Transplantation. Transplantation. 2020;104(11):2346-53. doi: 10.1097/tp.0000000000003157.

189. Wadhwani SI, Ge J, Gottlieb L, Lyles C, Beck AF, Bucuvalas J, et al. Racial/ethnic disparities in wait-list outcomes are only partly explained by socioeconomic deprivation among children awaiting liver transplantation. Hepatology. 2022;75(1):115-24. doi: 10.1002/hep.32106.

190. Wall LB, Wright M, Samora J, Bae DS, Steinman S, Goldfarb CA. Social Deprivation and Congenital Upper Extremity Differences-An Assessment Using PROMIS. J Hand Surg Am. 2021;46(2):114-8. doi: 10.1016/j.jhsa.2020.08.017.

191. Warner TD, Settersten RA, Jr. Why Neighborhoods (and How We Study Them) Matter for Adolescent Development. Adv Child Dev Behav. 2017;52:105-52. doi: 10.1016/bs.acdb.2016.10.003.

192. Wen M. Social Capital and Adolescent Substance Use: The Role of Family, School, and Neighborhood Contexts. J Res Adolesc. 2017;27(2):362-78. doi: 10.1111/jora.12299.

193. Wheeler DC, Boyle J, Nelson EJ. Modeling annual elevated blood lead levels among children in Maryland in relation to neighborhood deprivation. Sci Total Environ. 2022;805:150333. doi: 10.1016/j.scitotenv.2021.150333.

194. Wheeler DC, Boyle J, Raman S, Nelson EJ. Modeling elevated blood lead level risk across the United States. Sci Total Environ. 2021;769:145237. doi: 10.1016/j.scitotenv.2021.145237.

195. Wheeler DC, Jones RM, Schootman M, Nelson EJ. Explaining variation in elevated blood lead levels among children in Minnesota using neighborhood socioeconomic variables. Sci Total Environ. 2019;650(Pt 1):970-7. doi: 10.1016/j.scitotenv.2018.09.088.

196. Wheeler DC, Raman S, Jones RM, Schootman M, Nelson EJ. Bayesian deprivation index models for explaining variation in elevated blood lead levels among children in Maryland. Spat Spatiotemporal Epidemiol. 2019;30:100286. doi: 10.1016/j.sste.2019.100286.

197. White-Traut R, Norr KF, Fabiyi C, Rankin KM, Li Z, Liu L. Mother-infant interaction improves with a developmental intervention for mother-preterm infant dyads. Infant Behav Dev. 2013;36(4):694-706. doi: 10.1016/j.infbeh.2013.07.004.

198. Williamson AA, Gould R, Leichman ES, Walters RM, Mindell JA. Socioeconomic disadvantage and sleep in early childhood: Real-world data from a mobile health application. Sleep Health. 2021;7(2):143-52. doi: 10.1016/j.sleh.2021.01.002.

199. Winkelman AJ, Beller HL, Morgan KE, Corbett ST, Leroy SV, Noona SW, et al. Benefits and barriers to pediatric tele-urology during the COVID-19 pandemic. J Pediatr Urol. 2020;16(6):840.e1-.e6. doi: 10.1016/j.jpurol.2020.09.028.

200. Yaeger JP, Moore KA, Melly SJ, Lovasi GS. Associations of Neighborhood-Level Social Determinants of Health with Bacterial Infections in Young, Febrile Infants. J Pediatr. 2018;203:336-44.e1. doi: 10.1016/j.jpeds.2018.08.020.

201. Yao J, Perzynski AT, Tarabichi Y, Swarup N, Roy A. Socioeconomic Distress as a Predictor of Missed First Outpatient Newborn Visits. Cureus. 2021;13(3):e14132. doi: 10.7759/cureus.14132.

202. Yingling ME, Bell BA, Hock RM. Treatment Utilization Trajectories among Children with Autism Spectrum Disorder: Differences by Race-Ethnicity and Neighborhood. J Autism Dev Disord. 2019;49(5):2173-83. doi: 10.1007/s10803-019-03896-3.

203. Younan D, Tuvblad C, Li L, Wu J, Lurmann F, Franklin M, et al. Environmental Determinants of Aggression in Adolescents: Role of Urban Neighborhood Greenspace. J Am Acad Child Adolesc Psychiatry. 2016;55(7):591-601. doi: 10.1016/j.jaac.2016.05.002.

204. Yun I, Lee J. Neighborhood Disadvantage and Parenting: Behavioral Genetics Evidence of Child Effects. Int J Offender Ther Comp Criminol. 2016;60(13):1549-68. doi: 10.1177/0306624x15581451.

205. Zhang H, Lee ZX, White T, Qiu A. Parental and social factors in relation to child psychopathology, behavior, and cognitive function. Transl Psychiatry. 2020;10(1):80. doi: 10.1038/s41398-020-0761-6.

206. Zimmerman GM, Messner SF. Individual, family background, and contextual explanations of racial and ethnic disparities in youths' exposure to violence. Am J Public Health. 2013;103(3):435-42. doi: 10.2105/ajph.2012.300931.
